# Genome-wide association study of bipolar disorder in Canadian and UK populations corroborates disease loci including *SYNE1* and *CSMD1*

**DOI:** 10.1186/1471-2350-15-2

**Published:** 2014-01-04

**Authors:** Wei Xu, Sarah Cohen-Woods, Qian Chen, Abdul Noor, Jo Knight, Georgina Hosang, Sagar V Parikh, Vincenzo De Luca, Federica Tozzi, Pierandrea Muglia, Julia Forte, Andrew McQuillin, Pingzhao Hu, Hugh MD Gurling, James L Kennedy, Peter McGuffin, Anne Farmer, John Strauss, John B Vincent

**Affiliations:** 1 Dalla Lana School of Public Health, University of Toronto, Toronto, Canada; 2 MRC SGDP Centre, King’s College London, Institute of Psychiatry, De Crespigny Park, London SE5 8AF, UK; 3 Cancer Care Ontario, Toronto, Canada; 4 Neurogenetics Section, Campbell Family Mental Health Research Institute, Centre for Addiction and Mental Health (CAMH), R-32, 250 College Street, Toronto, ON M5T 1R8, Canada; 5 Department of Psychiatry, University of Toronto, Toronto, Canada; 6 The Institute of Medical Science, University of Toronto, Toronto, Canada; 7 Centre for Addiction and Mental Health (CAMH), Toronto, Canada; 8 GSK Research & Development, Medical Genetics, Clinical Pharmacology and Discovery Medicine, Via Fleming 4, Verona, Italy; 9 Exploratory Medicine & Early Development, NeuroSearch, Copenhagen, Denmark; 10 Molecular Psychiatry Laboratory, Mental Health Sciences Unit, Faculty of Brain Sciences, University College London, London, UK; 11 The Centre for Applied Genomics, The Hospital for Sick Children Research Institute, Toronto, Canada; 12 GSK Research & Development, Medical Genetics, Clinical Pharmacology and Discovery Medicine, Greenford Road, Greenford, Middlesex UB6 OHE, UK

**Keywords:** Case/control, Trio, Transmission disequilibrium test, Pathway analysis

## Abstract

**Background:**

Recently, genome-wide association studies (GWAS) for cases versus controls using single nucleotide polymorphism microarray data have shown promising findings for complex neuropsychiatric disorders, including bipolar disorder (BD).

**Methods:**

Here we describe a comprehensive genome-wide study of bipolar disorder (BD), cross-referencing analysis from a family-based study of 229 small families with association analysis from over 950 cases and 950 ethnicity-matched controls from the UK and Canada. Further, loci identified in these analyses were supported by pathways identified through pathway analysis on the samples.

**Results:**

Although no genome-wide significant markers were identified, the combined GWAS findings have pointed to several genes of interest that support GWAS findings for BD from other groups or consortia, such as at *SYNE1* on 6q25, *PPP2R2C* on 4p16.1, *ZNF659* on 3p24.3, *CNTNAP5* (2q14.3), and *CDH13* (16q23.3). This apparent corroboration across multiple sites gives much confidence to the likelihood of genetic involvement in BD at these loci. In particular, our two-stage strategy found association in both our combined case/control analysis and the family-based analysis on 1q21.2 (closest gene: sphingosine-1-phosphate receptor 1 gene, *S1PR1*) and on 1q24.1 near the gene *TMCO1*, and at *CSMD1* on 8p23.2, supporting several previous GWAS reports for BD and for schizophrenia. Pathway analysis suggests association of pathways involved in calcium signalling, neuropathic pain signalling, CREB signalling in neurons, glutamate receptor signalling and axonal guidance signalling.

**Conclusions:**

The findings presented here show support for a number of genes previously implicated genes in the etiology of BD, including *CSMD1* and *SYNE1*, as well as evidence for previously unreported genes such as the brain-expressed genes *ADCY2*, *NCALD*, *WDR60*, *SCN7A* and *SPAG16*.

## Background

Bipolar disorder (BD), also known as manic-depressive illness, is a chronic and devastating psychiatric condition, affecting 0.5-1.6% of the general population across their lifetime [1]. The frequency of hospitalization, psychological impairment, family devastation and suicidal behaviour make BD a major public health concern [2,3], with an estimated total annual societal cost of at least 45 billion dollars in North America [4]. It is characterized by the recurrence of manic and hypomanic episodes. Although the majority of BD sufferers experience a significant reduction in symptoms between episodes of illness, approximately 60% develop chronic interpersonal and occupational impairment, with the result of untreated illness usually generating major disability [1]. Comorbidity with other psychiatric illness such as alcohol or substance abuse may also exacerbate the long-term course of BD [1].

Family, twin and adoption studies provide strong evidence of the genetic predisposition to BD [5,6], with heritability estimates typically in the region of 80%. The recurrence risk in siblings of a BD proband is ~8% (corresponding to a sibling relative risk compared with the general population of λ_S_ ~ 8), and for monozygotic (identical) co-twins the risk is ~ 60%.

Identification of susceptibility genes for BD is the first step on a path toward improved understanding of the pathogenesis of mood disorders,with much to offer including (a) more effective and better targeted treatments, (b) earlier recognition of individuals at risk, and (c) improved understanding of environmental factors [7-9]. Linkage study findings support the view that no variation within a single gene can explain the majority of cases of BD, and demonstrates features that are typical in studies of complex genetic disorders, such as: (1) No finding replicates in all data sets, (2) Modest levels of statistical significance and estimated effect sizes, and (3) Chromosomal regions of linkage are typically broad (often > 20–30 cM).

Recent advances in density and speed of high-throughput single nucleotide polymorphism (SNP) genotyping along with a reduction in costs has provided researchers with an excellent opportunity to dissect the genetics of BD, under the hypothesis that common variants contribute to the disease. There has since been a wave of large genome-wide association studies (GWAS) of BD that have used high-density SNP microarrays to look for common shared genotypes and haplotypes. Although such studies seem to suffer from many of the same problems as the family-based whole genome scans performed using microsatellite markers, i.e. failure to replicate across different sample sets and a realization that much larger sample sizes are necessary, the use of standard SNPs and genotyping methodologies has allowed the pooling of data from large patient cohorts, and this has led to some exciting findings of possible susceptibility loci and genes such as *DGKH*, *CACNA1C* and *ANK3*[10] (reviewed in Lee et al., 2012).

In the current study, we utilized an alternative and, we believe, more efficient strategy, that used genotype data from more than 950 BD cases and 950 psychiatrically screened controls collected from two sites with identical ascertainment criteria and assessment methods - one in Canada, the other in the UK. As a source of independent validation, we have also analysed genome-wide genotype data from a Canadian cohort of small families.

## Methods

### Subject recruitment

For the total 1922 samples, there are 871 samples from Toronto constituting 431 cases (160 males and 271 females) and 440 controls (176 males and 264 females); there are 1051 samples from UK including 538 cases (180 males and 358 females) and 513 controls (192 males and 321 females). A breakdown of mean and median age at interview, age of onset (AOO), diagnostic subtype (BD I versus BD II), presence of psychotic symptoms, suicide attempt and family history of psychiatric disorders has been provided previously for both the Toronto and UK cohorts [11]. The 229 Toronto parent-offspring trio families, including 215 families with BD proband and both parents, and 14 families with BD proband and just 1 parent. Demographic information and ascertainment criteria for the family cohort have been reported previously [12].

From the CAMH, Toronto site BD individuals and unrelated healthy controls matched for age, gender and ethnicity were recruited. Inclusion criteria for patients: a) diagnosed with DSMIV/ICD 10 BD I or II; b) 18 years old or over; c) Caucasian, of Northern and Western European origin, and three out of four grandparents also N.W. European Caucasian. Exclusion criteria include: a) Use of intravenous drugs; b) Evidence of mental retardation; c) Related to an individual already in the study; d) Manias *that only* ever occurred in relation to or as a result of alcohol or substance abuse or dependence and/or medical illness; e) Have any manias as a result of a non-psychotropic substance. In this study, the SCAN interview (Schedule for Clinical Assessments in Neuropsychiatry) was used. SCAN was developed in the framework of the World Health Organisation (WHO) and the National Institutes of Health (NIH) Joint Project on Diagnosis and Classification of Mental Disorders Alcohol and Drug Related Problems [13]. Details on SCAN are available at http://apps.who.int/iris/handle/10665/40356.

Using both SCAN and case note review, each case was assigned DSM-IV and ICD 10 diagnoses by two independent team members with extensive diagnostic experience according to lifetime consensus best-estimate diagnosis [14]. Lifetime occurrence of psychiatric symptoms was also recorded using the OPCRIT checklist modified for use with mood disorders [15].

Similar methods and criteria were also used to collect a sample of 538 BD cases and 513 controls in London at the Institute of Psychiatry [16] (as described in Gaysina et al., 2010).

Our third sample is an independent BD cohort)t of 229 parent/affected offspring trio families also collected in the Toronto area. Methods included recruitment from hospital clinics and through advertising, SCID-I interviews, and best estimate consensus diagnosis [17,18].

Both studies were approved by local Research Ethics Committees (the CAMH Research Ethics Board (REB) in Toronto, and the College Research Ethics Committee (CREC) at King’s College London, and informed written consent was obtained from all participants.

### Genotyping

Genome-wide genotyping was performed for the Toronto and London case/control cohorts using the IlluminaSentrix Human Hap550 BeadChip (Illumina Inc., San Diego, CA, USA). Data was extracted by the Illumina® Beadstudio software from data files created by the IlluminaBeadArray reader. Mainly, these were genotyped by Illumina Inc. (San Diego, CA, USA) however 280 samples (97 cases and 183 controls) from the Toronto cohort were genotyped at the Genome Quebec facility. For the Toronto parent-offspring trio family cohort, Affymetrix 5.0 arrays (Affymetrix, Santa Clara, CA, USA) were used, and genotyped by the London Regional Genomics Centre (London, Ontario).

### Sample and SNP quality control

After genotyping, the discovery cohort samples were subject to a battery of a quality control (QC) tests. Reported and genetic gender were examined using X-chromosome linked SNPs. Relatedness between samples, sample contaminations, mis-identification and duplications were tested using genome-wide identity-by-descent (IBD) estimation; inconsistent samples were dropped from the analysis. Separate QC was applied on the validation cohort including the 229 Toronto parent-offspring trio families.

SNPs were subject to QC before analysis. Samples and markers with call rates below 95% were excluded from analysis. We removed SNPs with minor allele frequencies below 1%. To minimize genotyping errors we excluded SNPs with p-value <10^-5^ for HWE of control samples. PLINK software was used for quality control steps described above [19].

### Population stratification

Principal component analysis was conducted with EIGENSTRAT [20] on the discovery cohort with SNPs selected after QC filtering. To ensure the most homogeneous groups for association analysis, we excluded subjects with outliers defined by EIGENSTRAT [20]. Principal components (PCs) were selected based on analysis of the scree plot. For the genetic association analysis, the selected principal components were adjusted in the logistic regression model to correct for population stratification. We did not apply principle component analysis on the validation cohort since the family-based association tests are robust against population substructure [21].

### Genotype imputation

Since the discovery and validation datasets were genotyped on different GWAS SNP platforms, and the validation dataset has a smaller number of SNPs, genotypes of the SNPs that are not in the validation cohort were imputed. Beagle V3.3.1 [22] was used for the trio family imputation using a trio reference panel (HapMap3 Phasing Data: ftp://ftp.ncbi.nlm.nih.gov/hapmap//phasing/2009-02_phaseIII/HapMap3_r2/CEU/TRIOS/[23]) as this has better accuracy than imputation using a phased reference panel [22]. Individual genotypes with probability less than 0.90 were not included. Hidden Markov models (HMMs) were used for the imputation [22].

### Association analysis

The association analyses were first applied on the discovery cohort with only autosomal markers tested for association. Although we used an additive genetic model for primary analyses, we also explored dominant and recessive genetic models for sensitivity analysis. Logistic regression models were applied based on a genetic additive model. Odds ratios (OR) and 95% confidence intervals (CI) were estimated for the cases compared to the control group. The association, adjusting for principal components from the EIGENSTRAT analysis, was tested using multivariate logistic regression (SAS v9.2, Cary, NC). Association analysis on the validation cohort of trio families was performed using the transmission disequilibrium test (TDT) [24]. Power calculations for association analyses were performed using QUANTO [25,26].

### Exploratory analysis on combined discovery and validation datasets

Exploratory analysis was applied on the combined dataset of unrelated case control individuals and the trio family data. A hybrid method was applied to combine the distinct estimates from separate case control samples and parent-offspring trio families [27]. The estimates obtained from separate analyses are combined into an overall risk estimate and provided with the corresponding p-value. As an exploratory analysis, the combined analysis was applied on the SNPs that were nominally significant (p < 0.01) in both the discovery and validation analysis.

### Overlap of data with other published GWAS studies

Analysis of microarray genotype data for a subset of the cases/control cohort (483 cases, 462 controls) from the Institute of Psychiatry, London has been included in several published GWAS meta-analysis reports, including the Wellcome Trust Case Control Consortium (WTCCC) 2007, Scott et al., 2009, and Sklar et al., 2011 [28-30]. Data from a subset of the Canadian cohort (334 cases and 257 controls) were also included in the meta-analyses published by Scott et al., 2009 and Sklar et al., 2011, and in a locus-specific replication analysis locus by McMahon et al., 2010 (3p21.1) [31] and Francks et al., 2010 (19q13) [32]. Thus, the current study includes 55 cases and 49 controls from the London cohort and 97 cases and 183 controls from the Toronto cohort that do not overlap with these studies. GWAS for the small families does not overlap with any published genome-wide study, and pathway analysis on these cohorts has not been published previously.

### Pathway analysis

Our pathway analysis on the discovery cohort data followed that described by Beyene et al. [33]. For each SNP passing QC, we performed univariate SNP association analysis using logistic regression in PLINK. We selected SNPs that have nominal association (p < 0.01) from the discovery data association analysis. This includes 5111 SNPs.We obtained the nearest gene for each of the selected SNP from the Illumina SNP annotation file (HumanHap550Yv3_Gene_Annotation, available from icom.illumina.com) based on physical distance. The SNPs were mapped to 2155 genes. For each of the mapped genes, we obtained an aggregate summary measure based on individual values for SNPs assigned to this gene. Here we used the *maximum of absolute summary measure* over all SNPs mapped to the gene [33].

The aggregated summary measure was used to evaluate the significance of predefined pathways using Ingenuity Pathway Analysis software (IPA, version 11904312). Briefly, for a given pathway, statistical significance of the pathway enrichment is calculated using a Fisher's exact test based on the number of genes annotated, number of genes represented in the input dataset, and the total number of genes being assessed in the experiment. A pathway was deemed significant if the adjusted p-value of enrichment was ≤ 0.05 (adjusted for multiple comparisons using a Benjamini-Hochberg correction [34]).

## Results and discussion

To test the common variant hypothesis more comprehensively, we performed an unbiased genome-wide association study of common variation using the discovery dataset of 1922 case–control samples. Findings were validated using the independent family-based cohort. Quality-control (QC) procedures were applied to the 510,740 single nucleotide polymorphisms (SNPs) in the discovery dataset and 440,794 SNPs in the validation dataset.

### Population stratification

**Figure 1:**
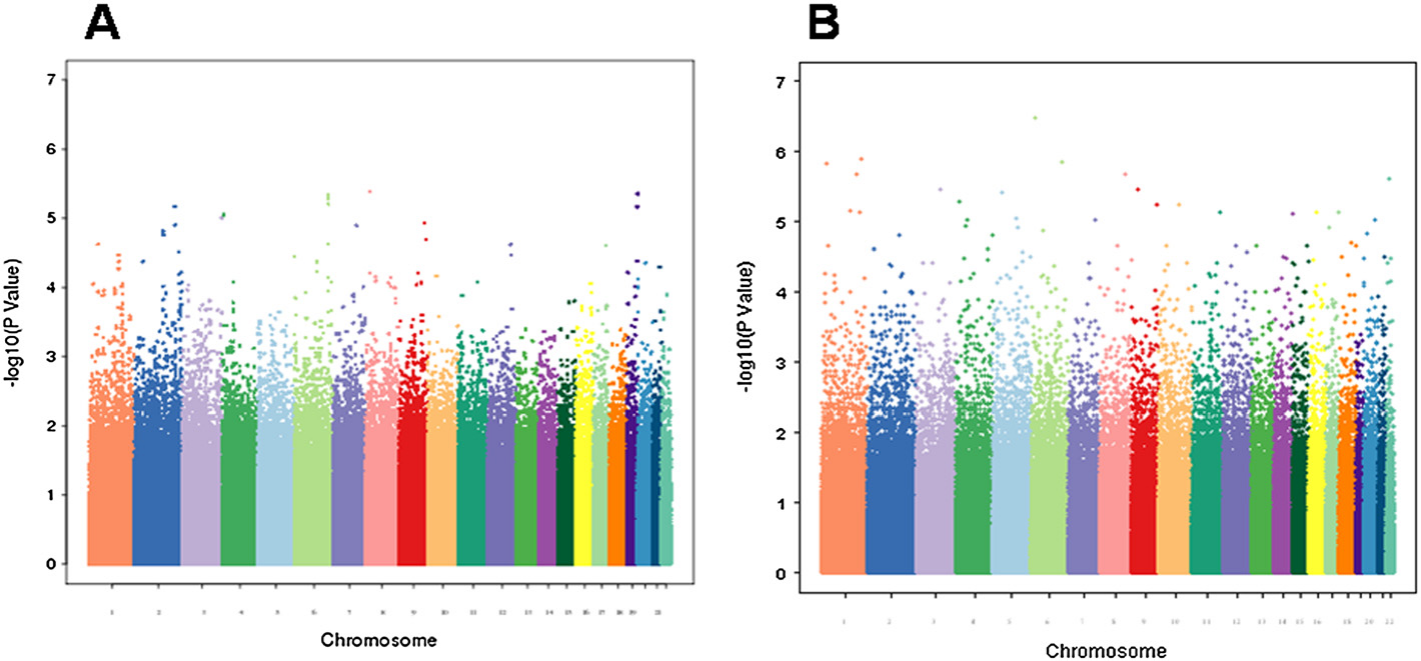
**A Manhattan plot is shown for A. the combined IoP/CAMH case/control cohort, and B. the CAMH small family sample, with –log10(P-value) plotted by genomic location for chromosomes 1–22.** SNPs from each chromosome are represented by different colors and ordered by physical positions.

**Figure 2:**
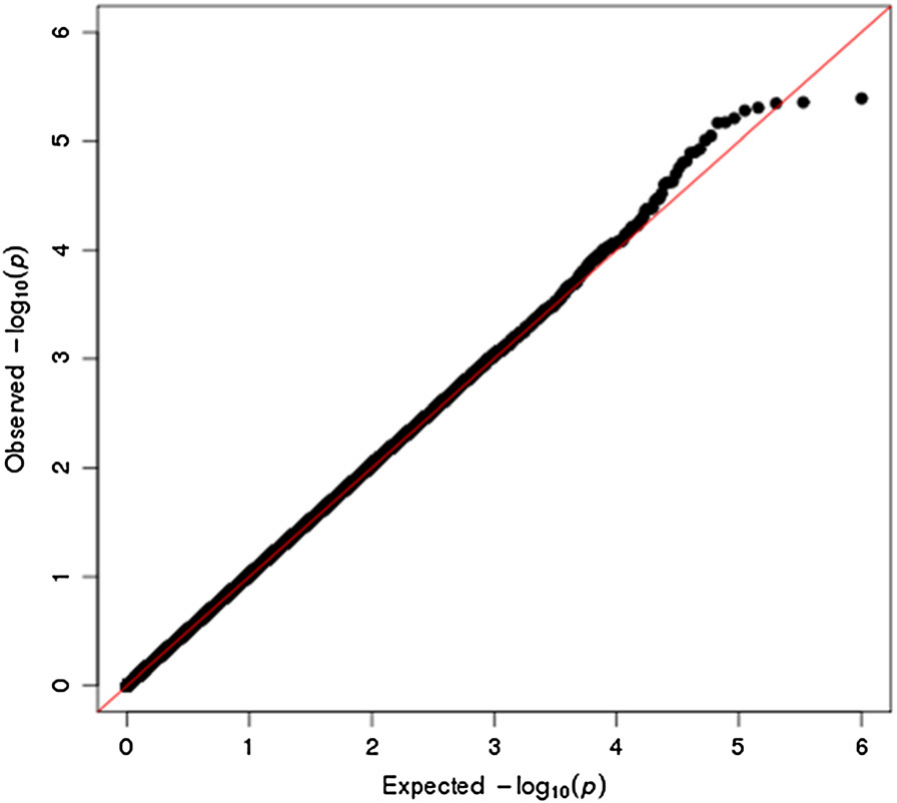
**Quantile-Quantile (Q-Q) plot of p-values for the case control dataset.** Note: The Q-Q plot measures deviation from the expected P-values. The diagonal (red) line represents the expected (null) distribution. The slight deviation of the observed values from expected values at the tail of the distribution is consistent with modest genetic effects.

After applying QC filters, 502,877 common autosomal SNPs remained in the discovery dataset and 346,565 common autosomal SNPs remained in the validation dataset. To account for possible population stratification, principal component analysis was undertaken with EIGENSTRAT [20]. Five subjects were identified as population outliers and excluded from the analysis. Three principal components were selected based on scree plot. Additional analyses for population stratification were undertaken with each of the genetic markers adjusting for the three principle components. The final datasets included 912 cases and 903 controls in the discovery dataset and 224 families (636 individuals) in the validation dataset. The average genotyping rate in the remaining individuals was 99.7%. The logistic regression model was used for association analyses in the discovery cohort. In the discovery dataset, none of the p-values met the stringent and perhaps overly conservative Bonferroni correction for genome-wide significance (Figure 1A). The distribution of p-values examined in the discovery dataset demonstrated a close match to that expected for a null distribution except at the extreme tail of low p-values (Figure 2).

### Discovery dataset analysis

**Figure 3:**
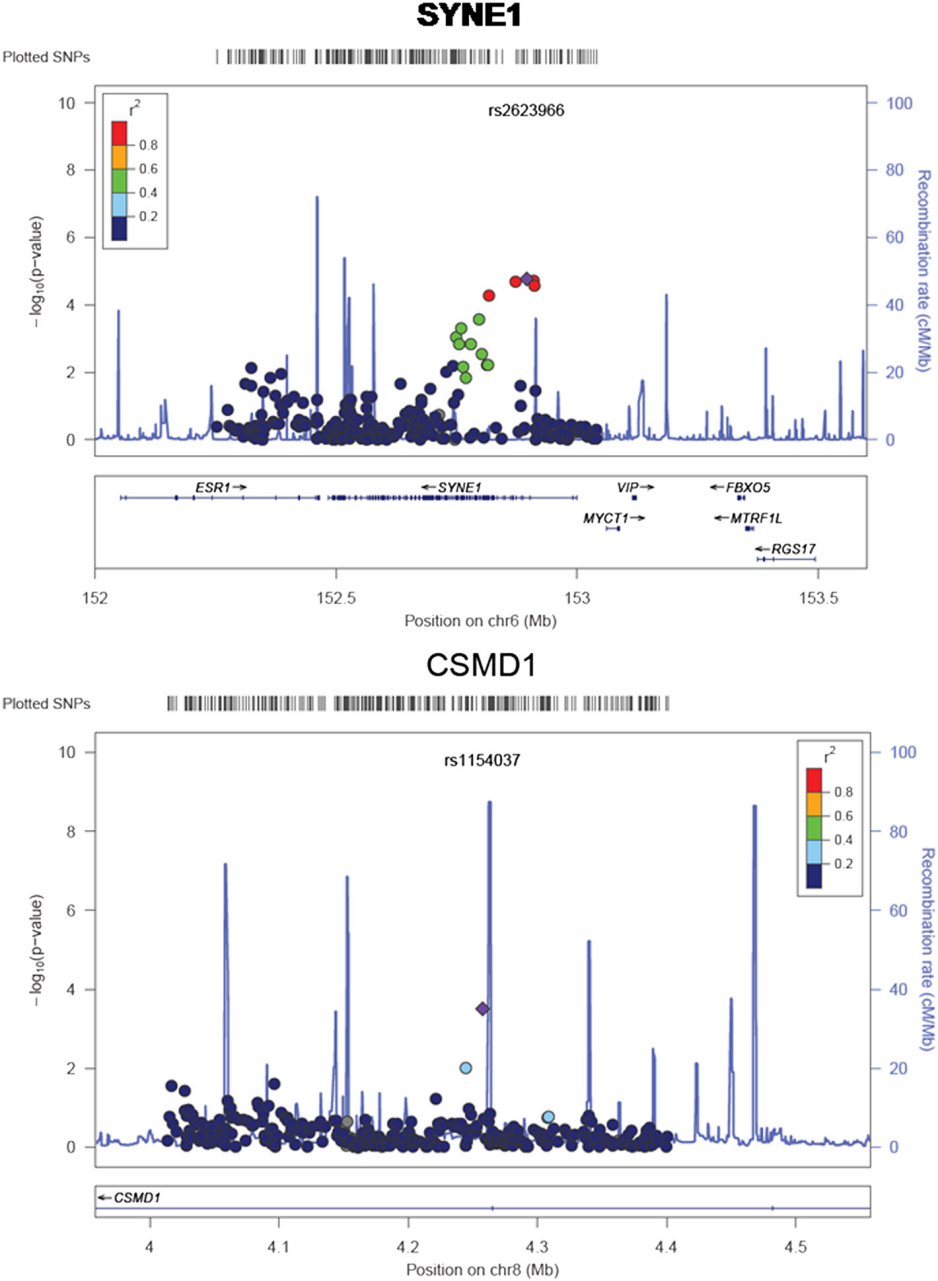
**Plots for association for combined IoP/CAMH cohorts across the *SYNE1*, and *CSMD1 *loci.** Probability of significance of association for SNPs passing quality control is shown as –log_10_ of the P-value on the left hand Y-axis. Recombination rate as estimated from HapMap (http://hapmap.ncbi.nlm.nih.gov/) is plotted in light blue. Chromosomal position is plotted according to NCBI build 36/Hg18. The SNP with the strongest evidence for association at each locus is shown as a blue diamond.Correlation of linkage disequilibrium between SNPs and the blue diamond SNP, r^2^, is colour-coded, red indicating stronger LD.

We computed the power of the 1815 samples in the discovery dataset. Given a prevalence of BD of 0.01, a SNP in LD (D' = 1) with a risk allele frequency 0.3, we have 76% power to detect significant association at p = 5.0E-7 under an additive model with strong effect size of OR 1.5. To detect an association with the same assumptions and at p = 5.0E-8 significance level, the statistical power is reduced to 0.61. With a moderate effect size of OR 1.3, the power to detect genome wide significant association (p < 5.0E-8) is very low. Despite no genome-wide significant association (p < 5.0E-8), 68 SNPs in our discovery dataset showed suggestive association with BD risk (p < 0.0001), many of which are replicating other GWAS findings for BD (Table 1 shows a subset of these SNPs with previous GWAS evidence for BD; the full set is given in Additional file 1). The most significant SNP was rs11787406, which is located just downstream of the gene *PRSS5* on 8p23.1 (p = 2.35E-6). Also, among the top 68 SNPs we see 6 SNPs within the gene *SYNE1* on 6q25, with lowest p = 3.02E-6 (plus a further 8 SNPs among the top 1000 SNPs; Additional file 1: Table S1; Figure 3). SNPs in this gene showed moderate association in the WTCCC study of ~2000 bipolar cases and ~3000 controls [28], with a genotypic p-value of 1.92E-05 for SNP rs2763025. Similarly, in a GWA meta-analysis [35] 14 SNPs within *SYNE1 *were identified with p value <9.0E-6. As the WTCCC study and Liu et al. meta-analyses included the case/control cohort from London, this cannot be presented as a completely independent observation, however the *SYNE1* SNP rs17082664 also showed suggestive association in a combined analysis of WTCCC plus STEP-UCL and ED-DUB-STEP2 datasets (p = 3.6E-6), with much of the signal coming from the STEP-UCL study, which on its own gives p = 3E-4 [36]. A single SNP in *ZNF659* on 3p24.3 was among the top 68 (with a further 4 SNPs among the top 1000; Additional file 1: Table S1). Sklar et al. [37] (no overlap with current datasets) also reported nominal association for this gene, with p = 3.25E-4 at rs259521. We also report 3 SNPs among the top 68 situated within the ZNF274 gene (rs4444432: p = 4.85E-6) on 19q13. However, this is someway distal to the nominal association for schizophrenia and psychosis reported by Francks et al. [32]. We also see suggestive association at rs4689410, within the *PPP2R2C* gene on 4p16.1 (p = 5.96E-6), which was previously reported to be associated to bipolar disorder [38,39].

**Table 1:** SNPs showing suggestive association (p < 0.0001) to BD in our combined (CAMH and IoP) GWAS

**Chr**	**SNP**	**bp**	**A1/A2**	**MAF**	**MAF_UK**	**MAF_CAN**	**P (UnAdj)**	**OR (UnAdj)**	**P (Adj)**	**OR (Adj)**	**P_UK**	**P_CAN**	**Gene**
1	rs2813164	96688031	C/T	0.3017	0.286	0.3195	3.75E-04	1.295	5.67E-05	1.349	0.07331	0.001371	Y0062^a^
3	rs11708571	21591377	G/A	0.3062	0.3163	0.2938	1.82E-04	0.7633	8.66E-05	0.7485	5.27E-05	0.2652	ZNF659^b,c,d^
4	rs4689410	6344204	A/G	0.3434	0.3569	0.3275	2.04E-05	0.7418	5.96E-06	0.7264	0.01966	0.0002366	PPP2R2C^e,f^
6	rs215006	152755628	A/G	0.2281	0.2095	0.2185	2.78E-04	1.335	7.83E-05	1.38	0.003807	0.001586	SYNE1^c^
6	rs214972	152775813	T/C	0.2271	0.2219	0.2327	4.73E-05	1.383	1.66E-05	1.419	0.003363	0.005299	SYNE1^c^
6	rs2623971	152831067	A/G	0.2107	0.2225	0.2348	1.86E-05	1.42	3.52E-06	1.481	0.004044	0.02257	SYNE1^c^
6	rs2623966	152853674	C/T	0.2122	0.2076	0.2173	1.50E-05	1.424	3.02E-06	1.481	0.002348	0.002308	SYNE1^c^
6	rs2141150	152868632	C/A	0.2138	0.2063	0.2155	1.73E-05	1.419	3.33E-06	1.476	0.002484	0.002721	SYNE1^c^
6	rs2695261	152869726	C/T	0.2069	0.2022	0.212	2.32E-05	1.417	4.12E-06	1.476	0.006699	0.001146	SYNE1^c^
9	rs7864144	101406038	G/A	0.1284	0.1294	0.1268	4.85E-04	0.7059	8.05E-05	0.6674	0.0008592	0.1271	GABBR2^f,h^
10	rs12773173	30011304	T/C	0.1028	0.1698	0.2155	1.26E-05	0.6902	6.56E-05	0.7054	0.02562	8.22E-05	SVIL^g^
19	rs1483651	58689834	C/A	0.1914	0.2857	0.2734	9.70E-06	1.389	7.55E-06	1.41	0.05476	1.10E-05	ZNF274^c^
19	rs4444432	58715538	G/T	0.2801	0.2868	0.2741	5.58E-06	1.402	4.85E-06	1.419	0.05396	4.94E-06	ZNF274^c^
19	rs7256349	58718269	A/G	0.281	0.3041	0.2839	4.09E-05	1.349	4.51E-05	1.359	0.03677	0.0001753	ZNF274^c^

^a^SNP or locus positive in [29].

^b^SNP or locus positive in [61].

^c^SNP or locus positive in [37].

^d^SNP or locus positive in [28].

^e^SNP or locus positive in [36].

^f^SNP or locus positive in Gurling-UCL top 1000 (*personal communication*).

^g^SNP or locus positive in [61].

^h^SNP or locus positive in [69].

Only SNPs included if located at genes with previous GWAS bipolar disorder associations. Chr = chromosome; SNP = single nucleotide polymorphism; bp = base pair (position from p telomere, according to NCBI36/hg18); A1 = allele 1; A2 = allele 2; MAF = minor allele frequency, (also for UK and Canadian (CAN) sets); P(UnAdj) = unadjusted p-value; OR(UnAdj) = unadjusted odds ratio; P(Adj) = adjusted p-value; OR(Adj) = adjusted odds ratio; Gene indicates SNP lies within a known gene. Full list of 68 SNPs showing suggestive association (p < 0.0001) is given in Additional file 1: Table S2.

In addition, a number of genes with no previous association to BD have multiple SNPs with suggestive association (Additional file 1: Table S4), including the brain-expressed genes, *ADCY2*, *NCALD*, *WDR60*, *SCN7A* and *SPAG16*.

### Validation dataset analysis

We computed the power of the TDT in 224 trio families in our validation sample. Given a prevalence of BD of 0.01, a SNP in LD (D' = 1) with a risk allele frequency 0.3, we have 82% power to detect association at the p = 0.05 significance level under an additive model with a strong effect of OR 1.5. To detect an association with a moderate effect size of OR 1.3, the statistical power reduce to 0.58. 132 SNPs in the validation dataset showed suggestive association (p < 0.0001), with the lowest p-value at rs16873052 on 6p24.1, uncorrected p = 3.19×10^-7^(Figure 1B). Other SNPs showing suggestive association included SNPs within known candidate genes for BD, such as *PDE4B* (p = 7.45×10^-5^). *PDE4B* encodes a phosphodiesterase that binds directly with DISC1, and is critical for cyclic adenosine monophosphate signalling, which is linked to learning, memory, and mood [40], and shows association with SCZ [41-44], and to some degree with BD [44]. SNPs were identified with suggestive association at a number of other genes with plausible biological arguments for involvement in, and/or previous associations to BD, such as *NRG3* (p = 5.53×10^-6^), *GAD2* (p = 2.21×10^-5^), *GRIK2* (p = 4.18×10^-5^), *GABRG3* (p = 3.83×10^-5^), and the synapse-associated protein 102 gene, *DLG3* (p = 5.31×10^-5^). In addition, a SNP in *ATP2A2*, the Darier disease gene (MIM#124200) also showed suggestive association (p = 2.67×10^-5^). Co-morbidity between Darier disease and BD has been known for some time [45], and linkage for BD to this locus has been reported in numerous studies [46-49]. Table 2 shows a subset of these 132 SNPs with previous evidence for BD or other neuropsychiatric disorders (the full set is given in Additional file 1).

**Table 2:** Selected SNPs (based on location within gene encoding protein of known function or disease association) showing suggestive association with TdT analysis (p < 0.0001) to BD in our validation dataset (small family cohort)

**Chr**	**SNP**	**bp**	**A1/A2**	**T/UT**	**TDT OR**	**P value**	**Chisq**	**Gene**	**Comments/MIM #**
1	rs575056	66522367	C/T	49/96	0.5104	9.5E-05	15.23	PDE4B	Protein interacts with DISC1, and is considered a candidate gene for BD and SCZ; MIM 600127
1	rs538336	66545251	G/A	63/116	0.5431	7.45E-05	15.69	PDE4B	MIM 600127
6	rs2852571	102108785	T/G	96/47	2.043	4.18E-05	16.79	GRIK2	Kainate glutamate receptor; homozygous mutations are associated with intellectual disability; MIM 138244
10	rs7074934	26571676	G/A	4/28	0.1429	2.21E-05	18	GAD2	Glutamate decarboxylase, responsible for converting L-glutamic acid to GABA; MIM 138275
10	rs17647803	83730201	G/A	11/45	0.2444	5.53E-06	20.64	NRG3	Member of neuregulin gene family, associated with BD and SCZ; MIM 605533
11	rs1893050	84305742	C/T	3/24	0.125	5.31E-05	16.33	DLG2	Post-synaptic density protein; MIM 603583
12	rs11065615	109214996	T/C	2/23	0.08696	2.67E-05	17.64	ATP2A2	MIM 107840; Mutations in this gene cause Darier disease (MIM 124200)
15	rs140673	25155075	C/A	8/35	0.2286	3.83E-05	16.95	GABRG3	GABA-A receptor; MIM 600233
16	rs7186123	81379253	G/T	20/54	0.3704	7.74E-05	15.62	CDH13	Cadherin gene associated with autism, also alcohol dependency; MIM 601364

The full list of 132 SNPs showing suggestive association (p < 0.0001) is given in Additional file 1: Table S3. Mendelian Inheritance in Man (MIM) number is included in comments, as a source of reference. Chr = chromosome; SNP = single nucleotide polymorphism; bp (base pair) indicates coordinates according to build hg18/NCBI36. Alleles are indicated in A1/A2. Transmitted (T) and untransmitted (UT) allele counts are given (T/UT), also odds ratio (OR) for the transmission disequilibrium test) TDT, and chisquare (Chisq) value.

### Exploratory analysis on combined discovery and validation datasets

Thirteen SNPs were nominal significant in the combined case/control discovery cohort and the trio validation cohort datasets with joint analysis p < 0.01 (Table 3). A sign test for the same direction of effect between discovery cohort and trio validation cohort was significant (p < 0.001). Several SNPs in this list overlap with candidate genes of interest in non-overlapping studies. These include SNP rs1154037 in *CSMD1*, for which the intronic SNP rs4875310 was suggestive significant in the Sklar et al. 2008 study (p = 3.74×10^-5^), as well as other SNPs at *CSMD1* in the Baum et al., 2008 study [50] (rs779105, NIMH cohort p = 0.0341, German cohort p = 0.0047; rs7812884, NIMH cohort p = 0.0012, German cohort p = 0.0103).

**Table 3:** Most significant overlap between discovery set (case/controls) and validation set (families)

**Chr**	**SNP**	**bp**	**MAF**	**MAF_UK**	**MAF_CAN**	**P-value:**	**P_UK**	**P_CAN**	**P-value:**	**P-value:**	**Gene**	**Function/domains?**
						**discovery**			**validation**	**joint**		
1	rs12564407	101581806	0.0372	0.0343	0.04089	5.02E-04	0.03818	0.0034	1.13E-05	0.00004	nr S1PR1	Sphingosine-1-phosphate receptor 1; MIM 601974
1	rs17452017	163951301	0.0708	0.07484	0.066	2.71E-04	0.002292	0.04094	1.00E-03	0.0003	nr TMCO1	Transmembrane and coiled-coil domains 1; MIM 614123
2	rs1564004	17962338	0.310	0.3233	0.295	7.85E-04	0.02767	0.009408	4.09E-03	0.0006	KCNS3	Potassium voltage-gated channel; MIM 603888
2	rs6437044	157852441	0.346	0.3413	0.3516	7.19E-04	0.05345	0.004589	4.18E-03	0.001	GALNT5	N-acetylgalactosaminyltransferase 5
2	rs7597971	166996418	0.314	0.303	0.3254	5.46E-04	0.1141	0.0007991	3.93E-03	0.0007	SCN7A	Sodium channel, voltage-gated, type VII, alpha; MIM 182392
5	rs4594899	1185216	0.446	0.4506	0.441	2.39E-04	0.07997	0.0007831	7.96E-03	0.002	nr SLC12A7	Potassium/chloride transporter MIM 604879
5	rs643502	140565698	0.420	0.4059	0.4357	9.18E-04	0.01301	0.03198	8.52E-03	0.001	nr PCDHB12	Protocadherin B12; MIM 606338
7	rs272706	24119442	0.488	0.4854	0.4906	3.91E-04	0.02933	0.005035	7.06E-03	0.007	nr NPY	Neuropeptide Y; MIM 162640
**8**	**rs1154037**	**4257636**	**0.0563**	0.05561	0.05744	**1.15E-03**	3.08E-05	0.6685	**6.04E-03**	**0.003**	**CSMD1**	**CUB and Sushi multiple domains 1; MIM 608397**
12	rs17041209	94684987	0.3276	0.3352	0.3189	5.86E-03	0.006487	0.2539	4.68E-03	0.002	NTN4	Promotes neurite elongation from olfactory bulb explants; MIM 610401
14	rs179524	30312602	0.223	0.2157	0.2307	3.33E-04	0.002542	0.04466	8.09E-03	0.001	nr SCFD1	Sec1 family domain containing 1
16	rs12443910	72711976	0.2943	0.316	0.2699	3.05E-03	0.002753	0.2762	9.82E-03	0.005	nr PSMD7	Proteasome 26S subunit, non-ATPase, 7; MIM 157970
20	rs13039978	57581000	0.0490	0.04626	0.05205	5.45E-04	0.1359	0.0007083	3.08E-03	0.001	nr PHACTR3	Phosphatase and actin regulator 3; MIM 608725
1	rs12564407	101581806	0.0372	0.0343	0.04089	5.02E-04	0.03818	0.0034	1.13E-05	0.00004	nr S1PR1	Sphingosine-1-phosphate receptor 1; MIM 601974

SNPs close to rs1154037, highlighted in bold, were positive in several other BD GWA studies [37,50], with no overlap in subjects. Where the SNP does not lie within a gene, the nearest coding gene is given (Nr = near). Chr = chromosome; SNP = single nucleotide polymorphism; bp (base pair) indicates coordinates according to build hg18/NCBI36; MAF = minor allele frequency, (also for UK and Canadian (CAN) sets). Mendelian Inheritance in Man (MIM) number, where available, is included with “function/domains?”, as a source of reference.

### Pathway analysis

Using the set of 2155 genes identified by our association analysis, pathway analyses were performed with IPA. From the pathway analysis of the 2155 genes (1956 of them were mapped to the IPA database) with nominal associations, 30 pathways were significantly enriched for at a Benjamini-Hochberg corrected p-value of 0.05 (see Table 4). Specific pathways involved in bipolar disorder (such as Neuropathic Pain Signaling, CREB Signaling in Neurons, etc.) were amongst the ones identified as most significant (Table 4). Consequently, these results suggest that the genes identified by our association analysis have a high degree of biological relevance.

**Table 4:** Canonical pathways enriched using 1956 genes from discovery set with adjusted p-value <0.05

**Ingenuity canonical pathways**	**-log(p)**	**P (adj)**	**Molecules**
Molecular Mechanisms of Cancer	4.54	0.003	RAP2B,PLCB2,JAK1,DIRAS3,PIK3R1,SMAD3,TAB2,CCND1,MYC,PTK2, CTNNA2,CAMK2D,RHOG,RHOB….
Neuropathic Pain Signaling In Dorsal Horn Neurons	4.42	0.003	PLCB2,CAMK4,GRIA1,PIK3R1,GNA11,TAC1,GNA14,GRIA4,TACR1,CAMK2D,PIK3C3,CREB1,CAMK1G …
Calcium Signaling	4.24	0.003	RAP2B,CAMK4,CALM,GRIA1,TNNT2,HDAC9,CREB5,GRIA4,CABIN1,NFAT5,CAMK2D….
Cellular Effects of Sildenafil (Viagra)	4.2	0.003	PLCB2,CAMK4,CALM1,CACNA1S,PDE3A,GNA11,GNA14,PDE4D….
Glutamate Receptor Signaling	4.15	0.003	GRIN2B,CALML5,CAMK4,CALM1,SLC17A6,GRID2,GRIA1,GRIK3,SLC1A3,GRIA4,GNG7,….
Axonal Guidance Signaling	4.11	0.003	PLCB2,PIK3R1,NTN1,PTK2,NCK2,EPHB1,SEMA3D,UNC5D….
ILK Signaling	3.99	0.004	MAP2K6,TMSL3,PPP2R2A,PIK3R1,DIRAS3,BMP2,HIF1A,CREB5,CCND1,PGF,PTK2,NCK2,MYC….
Nitric Oxide Signaling in the Cardiovascular System	3.49	0.0108	PDE2A,CALML5,GUCY1B2,CACNA1D,CAMK4,CALM1,GUCY1A3,CACNA1S,PIK3R1….
Gap Junction Signaling	3.27	0.0150	PLCB2,PIK3R1,GNA11,GNA14,TUBB2B,SP3,ADCY5,PIK3C3,PRKAR1B,CAV1,PRKCE,CTNNB1,ACTC1….
Role of NFAT in Cardiac Hypertrophy	3.23	0.0150	MAP2K6,PLCB2,CAMK4,CALM1,PIK3R1,GNA11,HDAC9,GNA14….
BMP signaling pathway	3.19	0.0150	CAMK4,GRB2,RRAS,BMP2,SMAD6,SMAD7,ZNF423,BMPR1B,JUN,PRKAR2B,MAP3K7,CREB1,PRKAR1B….
Dopamine-DARPP32 Feedback in cAMP Signaling	3.17	0.0150	PLCB2,CAMK4,CALM1,PPP2R2A,CACNA1S,GNA11,GNA14,CREB5,KCNJ4,ADCY5,CREB1,PRKAR1B…
CREB Signaling in Neurons	3.07	0.0161	PLCB2,CAMK4,CALM1,PIK3R1,GRIA1,GRID2,GNA11,GNA14,CREB5,GRIA4,GNG7,GRIK5,CAMK2D,GRID1….
N-Glycan Biosynthesis	3.04	0.0161	B4GALT4,ALG2,MGAT4A,MAN1C1,B4GALT1,ST6GAL2,MAN1A1,MGAT5,MAN1A2,MAN2A1,B4GALT5,FUT4
cAMP-mediated signaling	3.04	0.0161	AKAP12,CAMK4,CALM1,PTGER3,PDE3A,TAAR1,CREB5,CHRM3,CHRM1,PDE4D,HTR1B,CAMK2D,ADCY5….
Factors Promoting Cardiogenesis in Vertebrates	2.98	0.0174	SMAD2,FZD10,NOX4,TCF4,BMP2,FZD1,TCF3,FZD8,BMPR1B,MAP3K7 (includes EG:172842),PRKCE….
Melatonin Signaling	2.89	0.0201	MAP2K6,CALML5,PLCB2,CAMK4,MTNR1A,CALM1,GNA11,PLCL2,GNA14,CAMK2D,PRKAR2B,RORA….
Synaptic Long Term Potentiation	2.77	0.0235	GRIN2B,CALML5,PLCB2,CAMK4,CALM1,RRAS,GRIA1,GNA11,ITPR1,CREB5,GRIA4,GRIN3A,GRM7….
Amyotrophic Lateral Sclerosis Signaling	2.75	0.0235	GRIN2B,CACNA1D,GDNF,CAPN11,GRID2,CACNA1S,GRIA1,PIK3R1,GRIK3,GRIA4,GRIN3A,PGF,GRIK5….
Phospholipase C Signaling	2.73	0.0235	PLCB2,CAMK4,CALM1,DIRAS3,HDAC9,PLA2G2A,CREB5,GNG7,PLA2G4E,RHOG,NFAT5,RHOB,ADCY5….
PI3K Signaling in B Lymphocytes	2.73	0.0235	PLCB2,CAMK4,CALM1,PIK3R1,GNA11,GNA14,PTPRC,NFAT5,JUN,CAMK2D,CREB1,IRS2,PPP3CA….
Synaptic Long Term Depression	2.7	0.0241	PLCB2,PPP2R2A,GRID2,GRIA1,GNA11,PLA2G2A,GNA14,GRIA4,PLA2G4E,GRID1….
PPARα/RXRα Activation	2.6	0.0290	MAP2K6,PLCB2,NCOA6,SMAD3,GNA11,GNA14,IL6,CHD5,JUN,MAP3K7,ADCY5,PRKAR1B,PRKAA1….
TGF-β Signaling	2.44	0.0386	MAP2K6,SMAD2,INHA,GRB2,RRAS,BMP2,SMAD3,SMAD6,SMAD7,ZNF423,GSC,BMPR1B,PIAS4….
Protein Kinase A Signaling	2.44	0.0386	PLCB2,PDE3A,SMAD3,CREB5,PDE4D,NTN1,PTK2,CAMK2D,ADCY5,RYR3,PDE2A,YWHAE,PTCH1….
N-Glycan Degradation	2.38	0.0426	MANEA,GBA3,MAN1C1,SI,MAN1A1,HEXB,MAN1A2,MAN2A1
Regulation of IL-2 Expression in Activated and Anergic T Lymphocytes	2.36	0.0430	VAV2,SMAD2,CALML5,CAMK4,CALM1,GRB2,RRAS,SMAD3,MALT1,NFAT5,JUN,VAV3,ZAP70,MAPK10….
Wnt/β-catenin Signaling	2.32	0.0454	FZD10,TCF4,PPP2R2A,TLE1,FZD1,SOX13,CCND1,SOX2,MYC,SOX9,JUN,NLK,MAP3K7….
Agrin Interactions at Neuromuscular Junction	2.26	0.0498	RRAS,ITGA6,ITGA3,LAMC1,PTK2,JUN,NRG3,ERBB4,MUSK,LAMB1,MAPK10….
Colorectal Cancer Metastasis Signaling	2.25	0.0498	FZD10,TCF4,JAK1,PTGER3,PIK3R1,DIRAS3,MMP16,SMAD3,FZD1,IL6,CCND1,GNG7,BIRC5,PGF….

Ingenuity Pathway Analysis of the 1956 genes identified 30 significantly enriched pathways.

## Conclusions

Our GWA study presented here represents a multi-staged analysis, combining case/control genome-wide genotype data from two “sister” studies with parallel recruitment strategies and identical genotyping platforms as a discovery set, and using a family-based cohort consisting mainly of trios as a validation set. We reported our results by using suggestive significance (p < 0.0001) and nominal significance (p < 0.01). This is based on the concern that the SNPs across the genome are not independent, so a simple Bonferroni adjustment may be too conservative. Although relatively few results were suggestive significant in both discovery and validation sets, several of the overlapping SNPs are in genes of much interest for neuropsychiatric disease. One SNP in particular (rs1154037) is located within the third intron of the CUB and Sushi multiple domains 1 gene (*CSMD1*), which has been implicated by at least two further (non-overlapping) BD GWA studies [37,50]. *CSMD1*, which has also been associated with schizophrenia [51,52], is a complement control-related gene, and supports the theory of diminished activity of immunity-related pathways in the brain as a disease mechanism for psychiatric disorders including BD [53]. CSMD1 protein can inhibit the deposition of complement component C3 *in vitro*[54], and thus impaired function may lead to impaired regulation of the classical complement cascade. Alternatively, it is also known that proteins involved in regulating complement control can also regulate synaptic function [55,56]. Also of note, the neuropepetide Y gene, *NYP*, also identified by a nearby SNP in the joint discovery and validation analysis, was previously shown to be significantly down-regulated in the dorsolateral prefrontal cortex of psychosis patients [57], and in prefrontal cortex of BD subjects [58].

Analysis of associations from our discovery set shows strong support for the *SYNE1* locus (Table 1; Figure 3), albeit not at genome-wide significance levels. *SYNE1* has been implicated in a number of independent studies. Mutations of this gene are known as a cause for autosomal recessive spinocerebellar ataxia 8 (MIM 610743) and Emery-Dreifuss muscular dystrophy 4 (MIM 612998). *SYNE1* encodes a nesprin-1 component of a complex that links the cytoskeleton and nucleoskeleton (reviewed in [59]). However, several brain specific isoforms of rat Syne1 have been shown to localize to the postsynaptic side of synapses of glutamatergic neurons, and may be part of a mechanism of endocytosis of synaptic proteins, including glutamate receptors [60].

Our comparison with data from an independent BD GWAS from University College London showed joint suggestive significant loci at *CDH13*, *PPP2R2C* and *IGFBP7* (McQuillin and Gurling, *personal communication*). Comparison with other published GWA studies for bipolar disorder, excluding those with partial overlap of subjects, appears to corroborate several loci and candidate genes, including *CNTNAP5*[50,61], *ZNF804A*[62], *ZNF659*[37], *SORCS2*[50,63,64], and *ZNF536*[61]. A full list is provided in the Additional file 1: Table S2). *CNTNAP5*, encoding another neurexin-like protein, has also been linked with autism [65]. Interestingly, *CDH13* was also suggestive significant in our validation set (rs7186123; p = 7.74E-5), however the odds ratio suggests this allele as protective, whereas for the suggestive significant SNPs at *CDH13* in the discovery set the alleles appear mostly to be risk alleles (Additional file 1: Tables S1, S2 and S3).

Of the three zinc finger genes listed as loci showing suggestive significant association in our combined case/control study and in other bipolar GWA studies, little is known about the function, except for *ZNF536* on chromosome 19p13.3, which is highly expressed in the developing brain, and in cerebral cortex, hippocampus and hypothalamus and is believed to be a negative regulator of neuronal differentiation [66].

Suggestive association was seen at SNP rs4689410 within the gene *PPP2R2C* in our study (p = 5.96E-6). This gene has been previously reported to be associated with BD [38,39], and has also shown modest association in the UCL study, for SNP rs13122929 (p = 9.95E-4; McQuillin and Gurling, *personal communication*). Disruption of this gene may also be a cause of autosomal dominant intellectual disability (ID) [67]. This is one among a number of genes for which disruption may cause ID and for which common alleles may also be associated with risk for BD or SCZ (e.g. *ANK3, TCF4* and *NRXN1*).

Interestingly, a number of well established GWAS candidate genes are not represented among our top 1000 p-values, including *CACNA1C*, *ANK3* and *DGKH*[50,64], or ODZ4 [30]. This could reflect differences in the population in terms of heterogeneity of phenotype or ethnicity, or an issue of insufficient power to detect an effect, or effects due to differences in method of ascertainment. Conversely, a number of genes in our validation set show multiple SNPs with suggestive association that have not been reported elsewhere (Additional file 1: Table S4), including the brain-expressed genes, *ADCY2*, *NCALD*, *WDR60*, *SCN7A*, *SPAG16*. It will be of much interest to see whether support for these genes, for which no phenotype has previously been reported (Online Mendelian Inheritance in Man) [68], increases in BD meta-studies, once the sample size exceeds the tens of thousands.

In summary, the findings here support several key genetic associations to genes for BD, such as *CSMD1, SYNE1.*

## Competing interests

At the time of the study, authors FT, PM and JF were employees of GlaxoSmithKline (GSK). FT is currently affiliated with GSK. The other authors declare that they have no competing interests.

## Authors’ contributions

JBV, JS, JLK and WX conceived and designed the study. PM, FT designed the data collection with support for clinical data collection and analysis from PMc and AF. JF assisted with coordination of the sample and data collection. JS, SVP and GH assisted with the clinical data collection. WX and QC performed statistical procedures, with assistance in data analysis and interpretation from AN, JK, VD-L, AM and HMDG. JBV and WX drafted the manuscript with assistance from JK and SW-C. All authors read and approved the final manuscript.

## Pre-publication history

The pre-publication history for this paper can be accessed here:

http://www.biomedcentral.com/1471-2350/15/2/prepub

## Supplementary Material

Additional file 1: Table S1 SNPs from top 1000 from our combined CAMH/IoP GWAS for BPAD, for which at least one other non-overlapping GWAS also shows association at same gene. **Table S2**: Top 68 SNPs (showing suggestive association to BD: p < 0.0001) in our combined (CAMH and IoP) GWAS. **Table S3**. Top 132 SNPs (showing suggestive association to BD in our CAMH family cohort: p < 0.0001). **Table S4**: Listing suggestive significant genic SNPs for combined Toronto and London GWAS for which there are 4 or more suggestive significant SNPs among the top 1000, and for which no other positive reports have been published to date **Figure S1**: Scree plot of principal components (PCs) of the genotypes in the case–control samples. Click here for file
