# Supplementary material for: Genome-wide association study of bipolar disorder in Canadian and UK populations corroborates disease loci including SYNE1 and CSMD1
Source: BMC Med Genet. 2014 Jan 4;15:2. doi: 10.1186/1471-2350-15-2 (PMC3901032; doi:10.1186/1471-2350-15-2)

**Supplementary Table 1:** SNPs from top 1000 from our combined CAMH/IoP GWAS for BPAD, for which at least one other non-overlapping GWAS also shows association at same gene. For SNPs to be allocated within a gene, the SNP has to be either exonic or intronic. Where the SNP does not lie within a gene, it is assigned as intergenic and the nearest coding gene is given (nr= near)The full list of the top 1000 SNPs is available from the corresponding author.

| **Chr** | **Position, bp (hg18)** | **SNP** | **Adjusted *P* (ADD)** | **A1/A2** | **MAF** | **OR** | **Genes** | **SNP or Locus positive: References** |
| --- | --- | --- | --- | --- | --- | --- | --- | --- |
| 2 | 124933616 | rs2420864 | 5.45E-04 | T/C | 0.05339 | 0.5887 | CNTNAP5 | [29,50]# |
| 2 | 185464381 | rs17617913 | 7.46E-04 | C/T | 0.1266 | 1.419 | ZNF804A | [62] |
| 3 | 21566381 | rs11708571 | 8.66E-05 | G/A | 0.3062 | 0.7485 | ZNF659 | [28,36,37] |
| 3 | 21593524 | rs1457582 | 9.56E-04 | T/C | 0.4097 | 0.7996 | ZNF659 | [28,36,37] |
| 3 | 21596800 | rs12494223 | 1.44E-04 | T/C | 0.3022 | 0.7548 | ZNF659 | [28,36,37] |
| 3 | 21616961 | rs3860582 | 1.37E-04 | T/C | 0.3519 | 0.7617 | ZNF659 | [28,36,37] |
| 3 | 51348747 | rs9819195 | 1.09E-03 | G/A | 0.02046 | 0.4297 | DOCK3 | [50]* |
| 3 | 71040586 | rs6790743 | 1.66E-03 | C/A | 0.3284 | 0.8006 | Intergenic; nr FOXP1 (~300Kb) | [37] |
| 4 | 6454199 | rs4688994 | 2.27E-04 | T/C | 0.2446 | 0.7509 | PPP2R2C | $ |
| 4 | 6462276 | rs4689410 | 5.96E-06 | A/G | 0.3434 | 0.7264 | PPP2R2C | $ |
| 4 | 7538857 | rs4292336 | 1.48E-03 | G/A | 0.1581 | 0.7444 | SORCS2 | [50.63,64] |
| 4 | 57775899 | rs4865174 | 7.65E-04 | G/A | 0.1606 | 1.366 | IGFBP7 | [70]$ |
| 6 | 152800424 | rs214950 | 2.81E-04 | A/G | 0.2287 | 1.345 | SYNE1 | [28,36] |
| 6 | 152810736 | rs548978 | 1.40E-04 | G/A | 0.2292 | 1.364 | SYNE1 | [28,36] |
| 6 | 152847742 | rs215006 | 7.83E-05 | A/G | 0.2281 | 1.38 | SYNE1 | [28,36] |
| 6 | 152867927 | rs214972 | 1.66E-05 | T/C | 0.2271 | 1.419 | SYNE1 | [28,36] |
| 6 | 152923181 | rs2623971 | 3.52E-06 | A/G | 0.2107 | 1.481 | SYNE1 | [28,36] |
| 6 | 152945788 | rs2623966 | 3.02E-06 | C/T | 0.2122 | 1.481 | SYNE1 | [28,36] |
| 6 | 152960746 | rs2141150 | 3.33E-06 | C/A | 0.2138 | 1.476 | SYNE1 | [28,36] |
| 6 | 152961840 | rs2695261 | 4.12E-06 | C/T | 0.2069 | 1.476 | SYNE1 | [28,36] |
| 8 | 3387027 | rs922790 | 8.76E-04 | C/T | 0.4227 | 0.7941 | CSMD1 | [37,50] |
| 8 | 3395704 | rs10089209 | 1.01E-03 | T/C | 0.4413 | 0.7984 | CSMD1 | [37,50] |
| 8 | 3397152 | rs12056919 | 5.15E-04 | A/G | 0.4689 | 1.271 | CSMD1 | [37,50] |
| 8 | 3573418 | rs17063147 | 9.97E-04 | C/A | 0.1223 | 0.7162 | CSMD1 | [37,50] |
| 8 | 4257636 | rs1154037 | 7.50E-04 | C/T | 0.05629 | 0.5986 | CSMD1 | [37,50] |
| 8 | 56476497 | rs906168 | 7.57E-04 | T/C | 0.4255 | 0.8003 | XKR4 | [61] |
| 8 | 133192142 | rs2403733 | 1.99E-04 | G/A | 0.06788 | 0.6013 | intergenic, nr KCNQ3 | [37] |
| 8 | 133193525 | rs7842590 | 1.70E-04 | A/G | 0.06942 | 0.6008 | intergenic, nr KCNQ3 | [37] |
| 9 | 83062681 | rs1323771 | 3.40E-04 | A/G | 0.2302 | 0.747 | FRMD3 | [61] |
| 9 | 83079012 | rs10780579 | 3.85E-04 | T/C | 0.184 | 0.7287 | FRMD3 | [61] |
| 9 | 83081853 | rs10746698 | 4.55E-04 | T/C | 0.1843 | 0.7317 | FRMD3 | [61] |
| 9 | 83085154 | rs10117817 | 3.96E-04 | T/C | 0.1841 | 0.7292 | FRMD3 | [61] |
| 9 | 98485593 | rs7864144 | 8.05E-05 | G/A | 0.1284 | 0.6674 | GABBR2 | [61] |
| 10 | 397584 | rs3922851 | 3.43E-04 | A/G | 0.3692 | 0.7817 | DIP2C | [61] |
| 10 | 30051310 | rs12773173 | 6.56E-05 | T/C | 0.1914 | 0.7054 | SVIL | [69] |
| 13 | 26257496 | rs7333854 | 9.83E-04 | G/T | 0.09036 | 0.6741 | Intergenic; nr GPR12 | [69] |
| 13 | 100590460 | rs10508054 | 2.12E-03 | C/A | 0.09725 | 0.7002 | VGCNL1 | [37] |
| 16 | 7140564 | rs899305 | 3.38E-04 | C/T | 0.3099 | 1.303 | A2BP1 | [69] |
| 16 | 7149938 | rs8055744 | 6.19E-04 | A/G | 0.2125 | 1.334 | A2BP1 | [69] |
| 16 | 81326069 | rs6565063 | 2.08E-04 | C/T | 0.2191 | 1.354 | CDH13 | [70]$ |
| 16 | 81387022 | rs4782731 | 1.19E-03 | G/A | 0.1636 | 1.353 | CDH13 | [70]$ |
| 16 | 82129393 | rs11640984 | 1.61E-03 | C/A | 0.2284 | 1.29 | CDH13 | [70]$ |
| 16 | 82139566 | rs12448273 | 2.46E-04 | A/G | 0.2807 | 1.319 | CDH13 | [70]$ |
| 16 | 82140092 | rs11644289 | 9.83E-05 | C/T | 0.3116 | 1.331 | CDH13 | [70]$ |
| 16 | 82157898 | rs7201194 | 1.74E-04 | A/G | 0.3139 | 1.315 | CDH13 | [70]$ |
| 16 | 82162766 | rs11649622 | 7.23E-04 | A/G | 0.1944 | 0.7482 | CDH13 | [70]$ |
| 17 | 5212528 | rs7213832 | 9.74E-04 | G/A | 0.0937 | 0.6785 | RABEP1 | [61] |
| 19 | 35665223 | rs2866972 | 5.86E-04 | G/A | 0.1207 | 1.435 | ZNF536 | [61] |
| 19 | 35671807 | rs12610463 | 1.12E-03 | A/G | 0.08871 | 1.484 | ZNF536 | [61] |
| 19 | 35687595 | rs4805573 | 8.87E-04 | T/C | 0.07828 | 1.533 | ZNF536 | [61] |
| 19 | 35705601 | rs4805574 | 8.38E-04 | G/A | 0.08021 | 1.53 | ZNF536 | [61] |
| 19 | 35712242 | rs12609580 | 2.46E-04 | T/C | 0.08494 | 1.575 | ZNF536 | [61] |

# also in a SCZ linkage region

*also in a bipolar linkage region

$ SNP or locus positive in UCL-BD (McQuillin & Gurling, personal communication)

**Supplementary Table 2:** Top 68 SNPs (showing suggestive association to BD: p<0.0001) in our combined (CAMH and IoP) GWAS. Gene location of SNP is given, if located within a gene.

| **Chr** | **SNP** | **Position, bp (hg18)** | **A1/A2** | **MAF** | **HWE_*p**** | ***P*(unadj)** | **OR (unadj)** | ***P*(adj)** | **OR (adj)** | **Gene** |
| --- | --- | --- | --- | --- | --- | --- | --- | --- | --- | --- |
| 1 | rs3820588 | 43368906 | T/C | 0.2291 | 0.2979 | 2.51E-05 | 0.7158 | 2.12E-05 | 0.7039 | WDR65 |
| 1 | rs11210072 | 72998827 | C/T | 0.4281 | 0.07135 | 4.68E-04 | 1.265 | 7.46E-05 | 1.308 |  |
| 1 | rs12134034 | 73000541 | A/G | 0.4278 | 0.06118 | 4.12E-04 | 1.268 | 6.37E-05 | 1.312 |  |
| 1 | rs2422320 | 73005353 | C/T | 0.4278 | 0.06118 | 4.12E-04 | 1.268 | 6.37E-05 | 1.312 |  |
| 1 | rs11578058 | 170444787 | T/C | 0.1411 | 0.3934 | 1.30E-04 | 0.6925 | 6.46E-05 | 0.6715 | KLHL20 |
| 1 | rs10798301 | 170680989 | G/A | 0.2328 | 0.4365 | 1.82E-04 | 0.7444 | 8.77E-05 | 0.7303 | RC3H1 |
| 1 | rs6681627 | 170858399 | A/C | 0.1457 | 0.6343 | 1.23E-04 | 0.6952 | 5.03E-05 | 0.6735 |  |
| 1 | rs989423 | 171514180 | T/C | 0.2272 | 0.7258 | 1.40E-04 | 0.7388 | 4.17E-05 | 0.7189 | RABGAP1L |
| 1 | rs1328179 | 190484880 | T/C | 0.4447 | 0.1687 | 3.09E-05 | 1.322 | 6.52E-05 | 1.313 |  |
| 1 | rs2813164 | 195153065 | C/T | 0.3017 | 0.6762 | 3.75E-04 | 1.295 | 5.67E-05 | 1.349 |  |
| 1 | rs12022258 | 195163630 | C/T | 0.2446 | 0.7684 | 8.75E-05 | 1.355 | 7.32E-05 | 1.365 |  |
| 2 | rs10173568 | 36789310 | C/T | 0.4309 | 0.4219 | 1.05E-04 | 0.7707 | 3.07E-05 | 0.7529 |  |
| 2 | rs16822430 | 143981207 | C/T | 0.2296 | 0.2971 | 3.25E-05 | 1.392 | 1.76E-05 | 1.417 | ARHGAP15 |
| 2 | rs10048784 | 144001769 | T/C | 0.295 | 0.7313 | 3.65E-05 | 1.352 | 1.58E-05 | 1.38 | ARHGAP15 |
| 2 | rs4430884 | 144059361 | C/A | 0.227 | 0.3439 | 3.41E-05 | 1.392 | 1.69E-05 | 1.419 | ARHGAP15 |
| 2 | rs3821298 | 160112026 | G/T | 0.4515 | 0.5839 | 2.30E-05 | 1.327 | 8.90E-05 | 1.309 | BAZ2B |
| 2 | rs13409895 | 209791567 | A/G | 0.4375 | 0.3857 | 7.49E-06 | 0.7406 | 9.05E-06 | 0.7415 |  |
| 2 | rs4673558 | 211487403 | T/C | 0.2477 | 0.2815 | 1.80E-05 | 0.7181 | 1.26E-05 | 0.705 |  |
| 2 | rs6726093 | 228476856 | C/T | 0.2165 | 0.4177 | 5.68E-05 | 0.722 | 2.23E-05 | 0.7056 |  |
| 2 | rs9288627 | 228480606 | G/T | 0.1567 | 0.5724 | 1.30E-04 | 0.7037 | 5.11E-05 | 0.6862 |  |
| 2 | rs2037596 | 228492763 | A/G | 0.1788 | 0.4707 | 1.88E-04 | 0.7225 | 6.57E-05 | 0.7049 |  |
| 2 | rs2116397 | 235906555 | T/C | 0.2163 | 0.3865 | 1.37E-04 | 1.362 | 9.66E-05 | 1.38 |  |
| 2 | rs11680933 | 238386650 | G/T | 0.2355 | 0.6119 | 4.17E-05 | 1.38 | 3.50E-05 | 1.394 | LRRFIP1 |
| 2 | rs3769086 | 238413747 | A/G | 0.2354 | 0.6851 | 7.37E-05 | 1.366 | 5.98E-05 | 1.379 | LRRFIP1 |
| 2 | rs3820813 | 238413907 | C/T | 0.2336 | 0.76 | 9.05E-05 | 1.361 | 6.76E-05 | 1.38 | LRRFIP1 |
| 3 | rs12485389 | 16892557 | G/A | 0.0840 | 0.4257 | 7.11E-04 | 1.508 | 8.20E-05 | 1.64 | PLCL2 |
| 3 | rs11708571 | 21566381 | G/A | 0.3062 | 1 | 1.82E-04 | 0.7633 | 8.66E-05 | 0.7485 | ZNF385D/  ZNF659 |
| 3 | rs2410806 | 196401252 | A/G | 0.1096 | 1 | 8.18E-05 | 1.536 | 6.69E-06 | 1.644 | XXYLT1 |
| 4 | rs4689410 | 6462276 | A/G | 0.3434 | 0.777 | 2.04E-05 | 0.7418 | 5.96E-06 | 0.7264 | PPP2R2C |
| 6 | rs1418707 | 150800 | C/T | 0.1799 | 0.9192 | 1.92E-05 | 0.6884 | 3.01E-05 | 0.6895 |  |
| 6 | rs9385507 | 99343291 | T/C | 0.2003 | 0.2988 | 4.32E-05 | 0.7112 | 5.21E-05 | 0.7057 |  |
| 6 | rs9385517 | 99357637 | A/G | 0.2009 | 0.3469 | 3.29E-05 | 0.7077 | 3.73E-05 | 0.7019 |  |
| 6 | rs215006 | 152847742 | A/G | 0.2281 | 0.6809 | 2.78E-04 | 1.335 | 7.83E-05 | 1.38 | SYNE1 |
| 6 | rs214972 | 152867927 | T/C | 0.2271 | 0.8342 | 4.73E-05 | 1.383 | 1.66E-05 | 1.419 | SYNE1 |
| 6 | rs2623971 | 152923181 | A/G | 0.2107 | 0.09283 | 1.86E-05 | 1.42 | 3.52E-06 | 1.481 | SYNE1 |
| 6 | rs2623966 | 152945788 | C/T | 0.2122 | 0.2194 | 1.50E-05 | 1.424 | 3.02E-06 | 1.481 | SYNE1 |
| 6 | rs2141150 | 152960746 | C/A | 0.2138 | 0.1512 | 1.73E-05 | 1.419 | 3.33E-06 | 1.476 | SYNE1 |
| 6 | rs2695261 | 152961840 | C/T | 0.2069 | 0.1406 | 2.32E-05 | 1.417 | 4.12E-06 | 1.476 | SYNE1 |
| 6 | rs9458854 | 163938992 | T/C | 0.0388 | 0.5065 | 4.18E-05 | 0.4817 | 5.21E-05 | 0.4707 | QKI |
| 7 | rs11771168 | 113498012 | T/C | 0.2435 | 0.2745 | 1.12E-05 | 1.407 | 1.06E-05 | 1.405 | FOXP2 |
| 8 | rs6981847 | 10443943 | C/T | 0.1715 | 0.1091 | 7.24E-05 | 1.422 | 3.57E-05 | 1.464 | PRSS55 |
| 8 | rs11787406 | 10459617 | T/G | 0.1931 | 0.1384 | 2.18E-06 | 1.497 | 2.35E-06 | 1.519 |  |
| 8 | rs7005099 | 26215932 | T/C | 0.0333 | 0.4236 | 5.40E-05 | 0.4588 | 6.33E-05 | 0.4505 | PPP2R2A |
| 8 | rs10102339 | 27023405 | A/G | 0.3806 | 0.5594 | 5.09E-04 | 1.269 | 9.70E-05 | 1.313 |  |
| 8 | rs4734525 | 102387130 | T/C | 0.4854 | 0.4642 | 4.58E-05 | 0.7625 | 7.99E-05 | 0.7678 |  |
| 8 | rs4734194 | 110219847 | T/G | 0.4967 | 0.00024 | 8.78E-05 | 0.7701 | 6.65E-05 | 0.7715 |  |
| 8 | rs6469241 | 110223108 | C/T | 0.4967 | 0.00023 | 1.18E-04 | 0.7741 | 8.98E-05 | 0.7754 |  |
| 8 | rs16904075 | 130523936 | C/T | 0.0512 | 0.7884 | 1.22E-04 | 0.5533 | 5.83E-05 | 0.5296 |  |
| 9 | rs894962 | 91241255 | T/C | 0.486 | 0.5492 | 1.06E-04 | 0.7727 | 9.25E-05 | 0.7701 |  |
| 9 | rs7864144 | 98485593 | G/A | 0.1284 | 0.2364 | 4.85E-04 | 0.7059 | 8.05E-05 | 0.6674 | GABBR2 |
| 9 | rs17649473 | 122471429 | A/G | 0.0713 | 0.4211 | 4.30E-06 | 0.5445 | 1.04E-05 | 0.5491 | OR1B1 |
| 9 | rs507998 | 132360045 | A/G | 0.2512 | 0.1844 | 2.21E-04 | 0.7532 | 1.21E-05 | 0.7094 | C9orf171 |
| 10 | rs12773173 | 30051310 | T/C | 0.1914 | 0.6287 | 1.26E-05 | 0.6902 | 6.56E-05 | 0.7054 | SVIL |
| 11 | rs11022428 | 12572786 | T/C | 0.1351 | 0.617 | 8.85E-05 | 0.6814 | 7.03E-05 | 0.6702 |  |
| 11 | rs1783678 | 93713104 | G/A | 0.3777 | 0.1222 | 5.19E-05 | 1.32 | 5.32E-05 | 1.322 |  |
| 12 | rs1858886 | 109723276 | A/G | 0.4777 | 0.5896 | 4.12E-05 | 1.314 | 6.25E-05 | 1.313 |  |
| 12 | rs1476470 | 109729751 | T/C | 0.4788 | 0.5896 | 3.07E-05 | 1.32 | 4.48E-05 | 1.32 |  |
| 16 | rs12443555 | 80172870 | A/G | 0.0212 | 0.6115 | 5.72E-06 | 0.3172 | 8.44E-05 | 0.345 | CMIP |
| 16 | rs11644289 | 82140092 | C/T | 0.3116 | 0.5109 | 6.55E-05 | 1.332 | 9.83E-05 | 1.331 | CDH13 |
| 17 | rs11869073 | 64988408 | C/A | 0.1829 | 0.3645 | 5.45E-05 | 0.7057 | 2.52E-05 | 0.6911 | MAP2K6 |
| 19 | rs10402864 | 3991305 | G/A | 0.1558 | 0.7349 | 5.81E-05 | 0.6904 | 5.19E-05 | 0.683 |  |
| 19 | rs1483651 | 63381646 | C/A | 0.2801 | 0.9287 | 9.70E-06 | 1.389 | 7.55E-06 | 1.41 |  |
| 19 | rs4444432 | 63407350 | G/T | 0.281 | 1 | 5.58E-06 | 1.402 | 4.85E-06 | 1.419 | ZNF274 |
| 19 | rs7256349 | 63410081 | A/G | 0.2948 | 0.7976 | 4.09E-05 | 1.349 | 4.51E-05 | 1.359 | ZNF274 |
| 20 | rs6140884 | 916892 | T/C | 0.0951 | 1 | 1.41E-04 | 1.548 | 5.48E-05 | 1.611 | RSPO4 |
| 20 | rs6132109 | 1875515 | G/A | 0.2249 | 0.2554 | 1.88E-05 | 0.7106 | 9.01E-05 | 0.7288 |  |
| 20 | rs6060612 | 33853941 | A/G | 0.1017 | 0.8748 | 5.59E-04 | 0.6824 | 4.42E-05 | 0.6299 | PHF20 |
| 21 | rs2837475 | 40461958 | G/A | 0.2234 | 0.2058 | 1.11E-04 | 1.362 | 3.16E-05 | 1.401 | DSCAM |

**Supplementary Table 3.** Top 132 SNPs (showing suggestive association to BD in our CAMH family cohort: p<0.0001). Gene location of SNP is given, if located within a gene. T= number of transmitted alleles; UT = number of untransmitted alleles. TDT OR= odds ratio for transmission disequilibrium test.

| **Chr** | **SNP** | **Position, bp (hg18)** | **A1/A2** | **T** | **UT** | **TDT OR** | ***P*-value** | **Chisquare** | **Gene** |
| --- | --- | --- | --- | --- | --- | --- | --- | --- | --- |
| 1 | rs16851781 | 15743090 | T/C | 3 | 24 | 0.125 | 5.31E-05 | 16.33 | DNAJC16 |
| 1 | rs2064072 | 27978820 | C/T | 11 | 48 | 0.2292 | 1.46E-06 | 23.2 | STX12 |
| 1 | rs6670779 | 40430036 | T/C | 0 | 18 | 0 | 2.21E-05 | 18 | RLF |
| 1 | rs7538610 | 65162070 | G/A | 1 | 19 | 0.05263 | 5.7E-05 | 16.2 | JAK1 |
| 1 | rs575056 | 66522367 | C/T | 49 | 96 | 0.5104 | 9.5E-05 | 15.23 | PDE4B^a^ |
| 1 | rs538336 | 66545251 | G/A | 63 | 116 | 0.5431 | 7.45E-05 | 15.69 | PDE4B^a^ |
| 1 | rs17131451 | 71068814 | A/G | 3 | 23 | 0.1304 | 8.77E-05 | 15.38 |  |
| 1 | rs7518687 | 166899607 | A/T | 35 | 84 | 0.4167 | 7.06E-06 | 20.18 |  |
| 1 | rs3766084 | 167505182 | C/T | 7 | 31 | 0.2258 | 9.89E-05 | 15.16 | NME7 |
| 1 | rs16848204 | 199513781 | G/C | 5 | 35 | 0.1429 | 2.1E-06 | 22.5 |  |
| 1 | rs17017240 | 209384919 | G/A | 1 | 23 | 0.04348 | 7.1E-06 | 20.17 |  |
| 1 | rs1401319 | 222400733 | G/A | 5 | 36 | 0.1389 | 1.29E-06 | 23.44 | FBXO28 |
| 1 | rs4279792 | 230525587 | G/C | 7 | 32 | 0.2188 | 6.25E-05 | 16.03 |  |
| 2 | rs2012647 | 23086459 | C/T | 5 | 30 | 0.1667 | 2.38E-05 | 17.86 |  |
| 2 | rs10490354 | 74774269 | A/G | 1 | 18 | 0.05556 | 9.62E-05 | 15.21 |  |
| 2 | rs260632 | 108893401 | G/A | 22 | 59 | 0.3729 | 3.94E-05 | 16.9 | EDAR |
| 2 | rs17549841 | 111542860 | A/G | 19 | 54 | 0.3519 | 4.2E-05 | 16.78 | ACOXL |
| 2 | rs6437143 | 158739802 | A/G | 6 | 33 | 0.1818 | 1.54E-05 | 18.69 | CCDC148 |
| 2 | rs16847635 | 163489430 | T/C | 13 | 43 | 0.3023 | 0.000061 | 16.07 |  |
| 2 | rs13417240 | 172921262 | C/T | 3 | 24 | 0.125 | 5.31E-05 | 16.33 |  |
| 2 | rs6735700 | 213860696 | C/T | 7 | 31 | 0.2258 | 9.89E-05 | 15.16 | SPAG16 |
| 3 | rs3846013 | 27680337 | G/A | 0 | 17 | 0 | 3.74E-05 | 17 |  |
| 3 | rs1131960 | 69240784 | G/T | 0 | 17 | 0 | 3.74E-05 | 17 | LMOD3 |
| 3 | rs1274270 | 115812099 | T/C | 2 | 27 | 0.07407 | 3.44E-06 | 21.55 | ZBTB20 |
| 3 | rs6788209 | 169571766 | G/A | 21 | 2 | 10.5 | 7.44E-05 | 15.7 | EGFEM1P |
| 4 | rs16887512 | 12343369 | T/C | 4 | 31 | 0.129 | 5.02E-06 | 20.83 |  |
| 4 | rs1425328 | 20918048 | A/G | 18 | 51 | 0.3529 | 7.11E-05 | 15.78 | KCNIP4 |
| 4 | rs1479755 | 37106693 | T/C | 3 | 25 | 0.12 | 3.22E-05 | 17.29 |  |
| 4 | rs11735322 | 46164754 | C/T | 13 | 47 | 0.2766 | 1.14E-05 | 19.27 |  |
| 4 | rs1459481 | 58688061 | A/T | 95 | 43 | 2.209 | 9.58E-06 | 19.59 |  |
| 4 | rs4485795 | 73142416 | C/G | 0 | 16 | 0 | 6.33E-05 | 16 | NPFFR2^b^ |
| 4 | rs17012341 | 88202140 | A/T | 3 | 24 | 0.125 | 5.31E-05 | 16.33 | AFF1 |
| 4 | rs10023321 | 164940493 | T/G | 1 | 20 | 0.05 | 3.38E-05 | 17.19 | "MARCH1" |
| 4 | rs17046894 | 166765092 | G/A | 14 | 47 | 0.2979 | 2.39E-05 | 17.85 |  |
| 4 | rs7677236 | 189376252 | C/T | 28 | 71 | 0.3944 | 1.55E-05 | 18.68 |  |
| 5 | rs16903467 | 14448478 | G/A | 2 | 21 | 0.09524 | 7.44E-05 | 15.7 | TRIO |
| 5 | rs16898411 | 29036650 | C/G | 0 | 16 | 0 | 6.33E-05 | 16 |  |
| 5 | rs10038294 | 36949835 | G/A | 7 | 38 | 0.1842 | 3.82E-06 | 21.36 | NIPBL |
| 5 | rs7716014 | 65424071 | T/A | 9 | 36 | 0.25 | 5.7E-05 | 16.2 |  |
| 5 | rs11960716 | 81282298 | T/C | 6 | 30 | 0.2 | 6.33E-05 | 16 |  |
| 5 | rs35703899 | 113437540 | C/T | 34 | 77 | 0.4416 | 4.48E-05 | 16.66 |  |
| 5 | rs17141755 | 116796350 | C/A | 5 | 32 | 0.1562 | 9.05E-06 | 19.7 | BC045192 |
| 5 | rs6897843 | 119898382 | A/G | 3 | 27 | 0.1111 | 1.18E-05 | 19.2 | PRR16 |
| 5 | rs31874 | 140349502 | C/T | 102 | 53 | 1.925 | 8.29E-05 | 15.49 | PCDHA1-13 |
| 5 | rs6860892 | 143188422 | A/G | 29 | 71 | 0.4085 | 2.67E-05 | 17.64 |  |
| 5 | rs10477273 | 144728025 | A/C | 4 | 27 | 0.1481 | 3.61E-05 | 17.06 |  |
| 5 | rs17064692 | 174573344 | C/A | 69 | 28 | 2.464 | 3.14E-05 | 17.33 |  |
| 6 | rs16873052 | 12707322 | C/T | 1 | 29 | 0.03448 | 3.19E-07 | 26.13 |  |
| 6 | rs6908216 | 37365791 | T/G | 8 | 34 | 0.2353 | 6.02E-05 | 16.1 | TBC1D22B |
| 6 | rs2492922 | 42247701 | A/G | 17 | 50 | 0.34 | 5.54E-05 | 16.25 | GUCA1A |
| 6 | rs1329524 | 47798593 | T/C | 0 | 19 | 0 | 1.31E-05 | 19 |  |
| 6 | rs520570 | 74054537 | T/A | 7 | 32 | 0.2188 | 6.25E-05 | 16.03 | KHDC1 |
| 6 | rs9343332 | 76697270 | A/G | 2 | 21 | 0.09524 | 7.44E-05 | 15.7 | IMPG1 |
| 6 | rs4707111 | 85769987 | A/G | 37 | 79 | 0.4684 | 9.64E-05 | 15.21 |  |
| 6 | rs2852571 | 102108785 | T/G | 96 | 47 | 2.043 | 4.18E-05 | 16.79 | GRIK2^c^ |
| 6 | rs1726144 | 112076319 | A/G | 110 | 59 | 1.864 | 8.74E-05 | 15.39 |  |
| 6 | rs9495760 | 140919142 | C/G | 6 | 38 | 0.1579 | 1.41E-06 | 23.27 |  |
| 7 | rs736624 | 98446138 | C/T | 6 | 30 | 0.2 | 6.33E-05 | 16 | TRRAP |
| 7 | rs193858 | 105363793 | T/C | 5 | 29 | 0.1724 | 3.86E-05 | 16.94 | CDH28 |
| 7 | rs6969594 | 108724362 | C/T | 0 | 16 | 0 | 6.33E-05 | 16 |  |
| 7 | rs17161272 | 139283087 | G/A | 6 | 34 | 0.1765 | 9.55E-06 | 19.6 | TBXAS1 |
| 8 | rs17813827 | 541687 | T/G | 24 | 60 | 0.4 | 8.57E-05 | 15.43 |  |
| 8 | rs1471054 | 29125106 | A/T | 3 | 23 | 0.1304 | 8.77E-05 | 15.38 | KIF13B |
| 8 | rs907290 | 76377652 | G/A | 48 | 97 | 0.4948 | 4.72E-05 | 16.56 |  |
| 8 | rs16907590 | 81406740 | T/C | 4 | 28 | 0.1429 | 2.21E-05 | 18 |  |
| 8 | rs1459005 | 113984315 | G/A | 8 | 33 | 0.2424 | 9.45E-05 | 15.24 | CSMD3 |
| 8 | rs16889548 | 118361627 | A/G | 5 | 35 | 0.1429 | 2.1E-06 | 22.5 |  |
| 8 | rs7461611 | 133117801 | A/G | 38 | 82 | 0.4634 | 5.9E-05 | 16.13 | OC90 |
| 8 | rs6651493 | 145984729 | C/T | 1 | 20 | 0.05 | 3.38E-05 | 17.19 |  |
| 9 | rs1411792 | 18139174 | C/T | 26 | 72 | 0.3611 | 3.37E-06 | 21.59 |  |
| 9 | rs10984823 | 121863991 | A/G | 8 | 33 | 0.2424 | 9.45E-05 | 15.24 |  |
| 9 | rs6597678 | 133014292 | G/C | 2 | 26 | 0.07692 | 5.75E-06 | 20.57 | NUP214 |
| 10 | rs10906418 | 13673279 | A/G | 35 | 76 | 0.4605 | 9.96E-05 | 15.14 | PRPF18 |
| 10 | rs17140395 | 17327759 | A/G | 18 | 52 | 0.3462 | 4.83E-05 | 16.51 |  |
| 10 | rs11013266 | 23368344 | C/A | 10 | 37 | 0.2703 | 8.2E-05 | 15.51 |  |
| 10 | rs7074934 | 26571676 | A/G | 4 | 28 | 0.1429 | 2.21E-05 | 18 | GAD2^d^ |
| 10 | rs12245803 | 30441456 | T/A | 6 | 31 | 0.1935 | 3.96E-05 | 16.89 | KIAA1462 |
| 10 | rs17647803 | 83730201 | G/A | 11 | 45 | 0.2444 | 5.53E-06 | 20.64 | NRG3^e^ |
| 10 | rs17093782 | 117667495 | C/T | 0 | 17 | 0 | 3.74E-05 | 17 | ATRNL1 |
| 10 | rs2981434 | 123277693 | T/C | 3 | 23 | 0.1304 | 8.77E-05 | 15.38 | FGFR2 |
| 11 | rs16911839 | 12919698 | C/G | 17 | 49 | 0.3469 | 8.19E-05 | 15.52 | TEAD1 |
| 11 | rs1893050 | 84305742 | C/T | 3 | 24 | 0.125 | 5.31E-05 | 16.33 | DLG2^f^ |
| 11 | rs4268468 | 89525932 | T/C | 36 | 79 | 0.4557 | 6.08E-05 | 16.08 | NAALAD2 |
| 11 | rs602435 | 106908728 | T/C | 9 | 36 | 0.25 | 5.7E-05 | 16.2 | ALKBH8 |
| 11 | rs17118780 | 115194667 | A/G | 21 | 55 | 0.3818 | 9.62E-05 | 15.21 |  |
| 11 | rs11821204 | 123710871 | A/T | 3 | 28 | 0.1071 | 7.12E-06 | 20.16 |  |
| 11 | rs17118807 | 115217186 | T/C | 0 | 17 | 0 | 3.74E-05 | 17 |  |
| 12 | rs10877947 | 61733824 | C/A | 0 | 18 | 0 | 2.21E-05 | 18 |  |
| 12 | rs11056473 | 15468996 | C/T | 2 | 21 | 0.09524 | 7.44E-05 | 15.7 | PTPRO |
| 12 | rs1492336 | 39295452 | G/T | 49 | 16 | 3.062 | 4.26E-05 | 16.75 |  |
| 12 | rs932051 | 62081485 | A/G | 11 | 39 | 0.2821 | 7.5E-05 | 15.68 |  |
| 12 | rs7134603 | 106727070 | T/G | 1 | 18 | 0.05556 | 9.62E-05 | 15.21 |  |
| 12 | rs7307637 | 106847330 | G/A | 37 | 79 | 0.4684 | 9.64E-05 | 15.21 |  |
| 12 | rs11065615 | 109214996 | T/C | 2 | 23 | 0.08696 | 2.67E-05 | 17.64 | ATP2A2^g^ |
| 13 | rs17056765 | 37344768 | C/T | 0 | 18 | 0 | 2.21E-05 | 18 |  |
| 13 | rs11840233 | 83337446 | C/T | 1 | 18 | 0.05556 | 9.62E-05 | 15.21 |  |
| 14 | rs10151270 | 22442904 | G/C | 8 | 33 | 0.2424 | 9.45E-05 | 15.24 | RBM23 |
| 14 | rs12879582 | 42057616 | A/G | 9 | 35 | 0.2571 | 8.87E-05 | 15.36 |  |
| 14 | rs1954209 | 54053137 | A/G | 0 | 16 | 0 | 6.33E-05 | 16 | CGRRF1 |
| 14 | rs1882844 | 76402120 | C/T | 3 | 25 | 0.12 | 3.22E-05 | 17.29 | C14orf166B |
| 14 | rs7149343 | 67634464 | G/A | 11 | 41 | 0.2683 | 3.18E-05 | 17.31 | RAD51L1 |
| 14 | rs11160805 | 103937732 | A/G | 17 | 55 | 0.3091 | 7.52E-06 | 20.06 |  |
| 15 | rs140673 | 25155075 | C/A | 8 | 35 | 0.2286 | 3.83E-05 | 16.95 | GABRG3 |
| 15 | rs17432461 | 34962248 | T/C | 59 | 22 | 2.682 | 3.94E-05 | 16.9 | LOC145845 |
| 15 | rs496499 | 39185608 | T/C | 10 | 37 | 0.2703 | 8.2E-05 | 15.51 | INO80 |
| 15 | rs16961283 | 46716087 | G/C | 16 | 48 | 0.3333 | 6.33E-05 | 16 | FBN1 |
| 15 | rs6495504 | 78553680 | G/A | 1 | 18 | 0.05556 | 9.62E-05 | 15.21 | ARNT2 |
| 15 | rs2283436 | 87532780 | C/T | 21 | 55 | 0.3818 | 9.62E-05 | 15.21 | ABHD2 |
| 15 | rs11856465 | 91844229 | G/C | 0 | 18 | 0 | 2.21E-05 | 18 | AK094352 |
| 15 | rs190183 | 95347713 | G/T | 4 | 27 | 0.1481 | 3.61E-05 | 17.06 |  |
| 16 | rs16961083 | 13078286 | A/G | 1 | 20 | 0.05 | 3.38E-05 | 17.19 | SHISA9 |
| 16 | rs13331266 | 46719572 | G/C | 4 | 25 | 0.16 | 9.64E-05 | 15.21 | ABCC12 |
| 16 | rs3104794 | 51199461 | C/G | 3 | 28 | 0.1071 | 7.12E-06 | 20.16 |  |
| 16 | rs16953278 | 52847981 | A/G | 22 | 57 | 0.386 | 8.22E-05 | 15.51 |  |
| 16 | rs7186123 | 81379253 | G/T | 20 | 54 | 0.3704 | 7.74E-05 | 15.62 | CDH13^h^ |
| 17 | rs7214165 | 15779424 | G/T | 3 | 27 | 0.1111 | 1.18E-05 | 19.2 |  |
| 17 | rs9897202 | 72246937 | G/A | 1 | 23 | 0.04348 | 7.1E-06 | 20.17 | MFSD11 |
| 18 | rs532644 | 7506346 | T/C | 97 | 47 | 2.064 | 3.09E-05 | 17.36 |  |
| 18 | rs16977404 | 39062544 | T/C | 1 | 19 | 0.05263 | 5.7E-05 | 16.2 |  |
| 18 | rs7231636 | 52688262 | C/G | 3 | 26 | 0.1154 | 1.95E-05 | 18.24 | WDR7 |
| 18 | rs17058466 | 71734333 | T/C | 0 | 18 | 0 | 2.21E-05 | 18 |  |
| 20 | rs4816165 | 970483 | T/C | 61 | 113 | 0.5398 | 8.08E-05 | 15.54 |  |
| 20 | rs6077589 | 970779 | T/C | 52 | 101 | 0.5149 | 7.45E-05 | 15.69 |  |
| 20 | rs2014425 | 3164638 | A/G | 3 | 25 | 0.12 | 3.22E-05 | 17.29 | SLC4A11 |
| 20 | rs1569812 | 11004993 | G/C | 18 | 55 | 0.3273 | 1.49E-05 | 18.75 |  |
| 20 | rs238157 | 47289639 | T/C | 9 | 40 | 0.225 | 9.49E-06 | 19.61 | DDX27 |
| 21 | rs17231256 | 39370759 | T/C | 69 | 28 | 2.464 | 3.14E-05 | 17.33 |  |
| 22 | rs11090364 | 23731482 | A/G | 29 | 70 | 0.4143 | 3.78E-05 | 16.98 |  |
| 22 | rs8140359 | 24608694 | A/G | 2 | 21 | 0.09524 | 7.44E-05 | 15.7 | MYO18B |
| 22 | rs11090591 | 28707313 | C/A | 10 | 45 | 0.2222 | 2.37E-06 | 22.27 | MTMR3 |
| 22 | rs1013512 | 33683172 | T/C | 3 | 25 | 0.12 | 3.22E-05 | 17.29 |  |
| 22 | rs3021268 | 38734637 | A/G | 23 | 59 | 0.3898 | 7.02E-05 | 15.8 | FAM83F |

^a^protein interacts with DISC1, and is considered a candidate gene for BD and SCZ

^b^neuropeptide FF receptor 2 isoform 1

^c^Associated with autism/ID, also mania, and suicidal ideation during citalopram treatment of major depression

^d^responsible for catalyzing the production of gamma-aminobutyric acid from L-glutamic acid

^e^associated with BPAD and SCZ

^f^post-synaptic density protein, Chapsyn-110

^g^Mutations in this gene cause Darier-White disease

^h^associated with autism, also alcohol dependency

**Supplementary Table 4:** Listing suggestive significant genic SNPs for combined Toronto and London GWAS for which there are 4 or more suggestive significant SNPs among the top 1000, and for which no other positive reports have been published to date.

| **Chr** | **Position, bp (hg18)** | **SNP** | **MAF** | ***P*(unadj)** | **OR (unadj)** | ***P*(adj)** | **OR(adj)** | **GENES** |
| --- | --- | --- | --- | --- | --- | --- | --- | --- |
| 1 | 43300495 | rs639476 | 0.4144 | 0.000232 | 0.7794 | 0.000429 | 0.7879 | WDR65 |
| 1 | 43318601 | rs663824 | 0.3915 | 5.96E-05 | 0.7607 | 0.000116 | 0.7672 | WDR65 |
| 1 | 43357170 | rs11210815 | 0.2551 | 0.000733 | 0.7726 | 0.001196 | 0.7757 | WDR65 |
| 1 | 43368906 | rs3820588 | 0.2291 | 2.51E-05 | 0.7158 | 2.12E-05 | 0.7039 | WDR65 |
| 1 | 194906520 | rs12025074 | 0.2394 | 0.000413 | 1.318 | 0.00019 | 1.342 | NEK7 |
| 1 | 194944293 | rs6428445 | 0.2402 | 0.000368 | 1.32 | 0.000179 | 1.342 | NEK7 |
| 1 | 195093076 | rs4412625 | 0.2856 | 0.000876 | 1.28 | 0.000259 | 1.313 | NEK7 |
| 1 | 195116643 | rs10922418 | 0.2835 | 0.001194 | 1.271 | 0.000352 | 1.305 | NEK7 |
| 2 | 143929311 | rs10803491 | 0.2788 | 0.000989 | 1.277 | 0.000522 | 1.303 | ARHGAP15 |
| 2 | 143952151 | rs11680245 | 0.2703 | 0.000278 | 1.314 | 0.000117 | 1.345 | ARHGAP15 |
| 2 | 143981207 | rs16822430 | 0.2296 | 3.25E-05 | 1.392 | 1.76E-05 | 1.417 | ARHGAP15 |
| 2 | 144001769 | rs10048784 | 0.295 | 3.65E-05 | 1.352 | 1.58E-05 | 1.38 | ARHGAP15 |
| 2 | 144059361 | rs4430884 | 0.227 | 3.41E-05 | 1.392 | 1.69E-05 | 1.419 | ARHGAP15 |
| 2 | 144063279 | rs13004397 | 0.2694 | 0.000371 | 1.306 | 0.000123 | 1.341 | ARHGAP15 |
| 2 | 144097276 | rs12615880 | 0.2734 | 0.000368 | 1.305 | 0.000176 | 1.326 | ARHGAP15 |
| 2 | 167105429 | rs6738031 | 0.3134 | 0.000589 | 0.7816 | 0.001265 | 0.7927 | SCN7A |
| 2 | 167113679 | rs7597971 | 0.3138 | 0.000546 | 0.7806 | 0.00117 | 0.7912 | SCN7A |
| 2 | 167148654 | rs12475889 | 0.3737 | 0.000266 | 0.775 | 0.000361 | 0.7774 | SCN7A |
| 2 | 167159592 | rs7565062 | 0.3114 | 0.000654 | 0.782 | 0.001399 | 0.7927 | SCN7A |
| 2 | 167168148 | rs1172384 | 0.3121 | 0.000701 | 0.7841 | 0.001487 | 0.7943 | SCN7A |
| 2 | 214675366 | rs7561066 | 0.3047 | 0.000289 | 1.3 | 0.000462 | 1.286 | SPAG16 |
| 2 | 214684205 | rs17761193 | 0.1789 | 0.000601 | 1.353 | 0.00211 | 1.31 | SPAG16 |
| 2 | 214697097 | rs1510548 | 0.2647 | 0.000902 | 1.285 | 0.001005 | 1.28 | SPAG16 |
| 2 | 214710493 | rs4673807 | 0.3983 | 0.000643 | 1.261 | 0.00071 | 1.26 | SPAG16 |
| 2 | 214767405 | rs1030143 | 0.185 | 0.000508 | 1.348 | 0.001394 | 1.313 | SPAG16 |
| 2 | 214793718 | rs10514629 | 0.141 | 0.00046 | 1.4 | 0.001906 | 1.349 | SPAG16 |
| 2 | 214793950 | rs16851487 | 0.1565 | 0.00026 | 1.399 | 0.001194 | 1.352 | SPAG16 |
| 2 | 214878030 | rs11887857 | 0.1414 | 0.001161 | 1.365 | 0.000646 | 1.394 | SPAG16 |
| 2 | 214893259 | rs6435818 | 0.1414 | 0.001161 | 1.365 | 0.000797 | 1.389 | SPAG16 |
| 2 | 238254864 | rs4638738 | 0.2018 | 0.000485 | 1.337 | 0.001024 | 1.319 | LRRFIP1 |
| 2 | 238276742 | rs3751112 | 0.1799 | 0.001342 | 1.322 | 0.004075 | 1.289 | LRRFIP1 |
| 2 | 238386650 | rs11680933 | 0.2355 | 4.17E-05 | 1.38 | 3.5E-05 | 1.394 | LRRFIP1 |
| 2 | 238405413 | rs2047169 | 0.23 | 0.000183 | 1.345 | 0.000126 | 1.362 | LRRFIP1 |
| 2 | 238412236 | rs1565853 | 0.4493 | 0.000215 | 1.281 | 0.000364 | 1.275 | LRRFIP1 |
| 2 | 238413747 | rs3769086 | 0.2354 | 7.37E-05 | 1.366 | 5.98E-05 | 1.379 | LRRFIP1 |
| 2 | 238413907 | rs3820813 | 0.2336 | 9.05E-05 | 1.361 | 6.76E-05 | 1.38 | LRRFIP1 |
| 3 | 81806055 | rs3772902 | 0.486 | 6.08E-05 | 0.7659 | 0.000577 | 0.7917 | GBE1 |
| 3 | 81811207 | rs1375086 | 0.4569 | 0.000124 | 0.7739 | 0.00104 | 0.801 | GBE1 |
| 3 | 81845477 | rs7620240 | 0.3385 | 0.000498 | 1.277 | 0.00117 | 1.263 | GBE1 |
| 3 | 81860370 | rs2594547 | 0.4567 | 7.65E-05 | 0.7678 | 0.000678 | 0.7949 | GBE1 |
| 3 | 81894474 | rs3860595 | 0.3678 | 0.001474 | 1.245 | 0.002935 | 1.231 | GBE1 |
| 5 | 7717530 | rs4289537 | 0.3526 | 0.000242 | 1.291 | 0.000492 | 1.272 | ADCY2 |
| 5 | 7718637 | rs2973331 | 0.3514 | 0.000195 | 1.296 | 0.000458 | 1.275 | ADCY2 |
| 5 | 7770396 | rs6878196 | 0.4058 | 0.000698 | 0.795 | 0.00176 | 0.8106 | ADCY2 |
| 5 | 7790474 | rs12189243 | 0.46 | 0.000714 | 1.253 | 0.001734 | 1.226 | ADCY2 |
| 5 | 7802240 | rs12186377 | 0.3693 | 0.000253 | 1.287 | 0.000507 | 1.269 | ADCY2 |
| 6 | 3201894 | rs4445096 | 0.1642 | 0.001477 | 0.7513 | 0.004136 | 0.7716 | PSMG4 |
| 6 | 3204714 | rs11242834 | 0.1155 | 0.000472 | 0.6937 | 0.000811 | 0.7032 | PSMG4 |
| 6 | 3207500 | rs4602754 | 0.1744 | 0.000116 | 0.7125 | 0.000794 | 0.7433 | PSMG4 |
| 6 | 3207522 | rs4421240 | 0.1744 | 0.000116 | 0.7125 | 0.000794 | 0.7433 | PSMG4 |
| 6 | 3211142 | rs4959793 | 0.1251 | 3.68E-05 | 0.6584 | 0.000149 | 0.6792 | PSMG4 |
| 6 | 3212709 | rs9328157 | 0.1251 | 3.68E-05 | 0.6584 | 0.000149 | 0.6792 | PSMG4 |
| 6 | 3215577 | rs1127473 | 0.07163 | 0.000962 | 0.6506 | 0.002483 | 0.6742 | PSMG4 |
| 6 | 3217806 | rs9501975 | 0.07163 | 0.000373 | 0.6286 | 0.001102 | 0.6514 | PSMG4 |
| 6 | 5122037 | rs416803 | 0.249 | 0.000903 | 0.7747 | 0.000894 | 0.7697 | LYRM4 |
| 6 | 5122984 | rs10458086 | 0.4763 | 9.05E-05 | 0.7704 | 0.000263 | 0.7793 | LYRM4 |
| 6 | 5125887 | rs445711 | 0.2485 | 0.001168 | 0.7788 | 0.001183 | 0.7744 | LYRM4 |
| 6 | 5129492 | rs419778 | 0.2391 | 0.00059 | 0.7649 | 0.00072 | 0.7636 | LYRM4 |
| 6 | 5134726 | rs17139748 | 0.2375 | 0.001012 | 0.7734 | 0.001095 | 0.7703 | LYRM4 |
| 6 | 5159982 | rs4246073 | 0.2394 | 0.001193 | 0.7768 | 0.001369 | 0.7752 | LYRM4 |
| 6 | 5183035 | rs2773294 | 0.2576 | 0.001153 | 0.781 | 0.001621 | 0.7826 | LYRM4 |
| 7 | 158210118 | rs10275341 | 0.1628 | 0.000151 | 1.411 | 0.000271 | 1.402 | WDR60 |
| 7 | 158210355 | rs9654726 | 0.1612 | 0.000138 | 1.414 | 0.00024 | 1.407 | WDR60 |
| 7 | 158211060 | rs10279428 | 0.1612 | 0.000147 | 1.412 | 0.000254 | 1.405 | WDR60 |
| 7 | 158221422 | rs6957744 | 0.161 | 0.000164 | 1.409 | 0.00029 | 1.4 | WDR60 |
| 7 | 158227386 | rs12154715 | 0.1617 | 0.00015 | 1.411 | 0.000261 | 1.402 | WDR60 |
| 8 | 103014039 | rs517811 | 0.3802 | 0.001183 | 0.8009 | 0.001368 | 0.7982 | NCALD |
| 8 | 103020312 | rs7003289 | 0.4302 | 0.000821 | 0.7988 | 0.001294 | 0.7991 | NCALD |
| 8 | 103022075 | rs2119226 | 0.4829 | 0.001482 | 0.8095 | 0.001461 | 0.8019 | NCALD |
| 8 | 103035324 | rs4145554 | 0.4148 | 0.001093 | 1.247 | 0.002117 | 1.237 | NCALD |
| 8 | 103050229 | rs4734606 | 0.4157 | 0.001296 | 1.242 | 0.003033 | 1.226 | NCALD |
| 8 | 103089567 | rs1351063 | 0.4569 | 0.000552 | 0.7921 | 0.002153 | 0.8141 | NCALD |
| 8 | 103106243 | rs1269713 | 0.249 | 0.000684 | 0.7701 | 0.001242 | 0.7762 | NCALD |
| 8 | 110191315 | rs4129682 | 0.4617 | 0.000659 | 0.7969 | 0.000591 | 0.7965 | TRHR/NUDCD1 |
| 8 | 110219847 | rs4734194 | 0.4967 | 8.78E-05 | 0.7701 | 6.65E-05 | 0.7715 | TRHR/NUDCD1 |
| 8 | 110223108 | rs6469241 | 0.4967 | 0.000118 | 0.7741 | 8.98E-05 | 0.7754 | TRHR/NUDCD1 |
| 8 | 110371223 | rs2980619 | 0.4768 | 0.001089 | 1.245 | 0.000699 | 1.256 | TRHR/NUDCD1 |
| 18 | 55285808 | rs585632 | 0.4835 | 0.000269 | 0.7847 | 0.000762 | 0.7969 | CCBE1 |
| 18 | 55288238 | rs644856 | 0.4642 | 0.000162 | 0.7777 | 0.000551 | 0.7931 | CCBE1 |
| 18 | 55293760 | rs656750 | 0.3964 | 0.000142 | 0.7721 | 0.000581 | 0.7899 | CCBE1 |
| 18 | 55297334 | rs668432 | 0.3336 | 0.001359 | 1.253 | 0.001935 | 1.248 | CCBE1 |
| 18 | 55297475 | rs2013210 | 0.3199 | 0.001044 | 1.265 | 0.001474 | 1.263 | CCBE1 |
| 18 | 55301148 | rs619037 | 0.2996 | 0.001389 | 1.261 | 0.00198 | 1.255 | CCBE1 |
| 18 | 55311367 | rs667482 | 0.4101 | 0.000337 | 0.7848 | 0.001136 | 0.801 | CCBE1 |
| 19 | 55462998 | rs8112504 | 0.2661 | 1.15E-05 | 0.7185 | 0.000103 | 0.7476 | MYH14 |
| 19 | 55463134 | rs8112720 | 0.2649 | 1.64E-05 | 0.7219 | 0.000138 | 0.7508 | MYH14 |
| 19 | 55463421 | rs3745504 | 0.4558 | 2.88E-05 | 0.7561 | 0.000235 | 0.7803 | MYH14 |
| 19 | 55464946 | rs1670725 | 0.1567 | 6.09E-05 | 0.6919 | 0.000726 | 0.7332 | MYH14 |

**Supplementary Figure 1:** Scree plot of principal components (PCs) of the genotypes in the case-control samples.


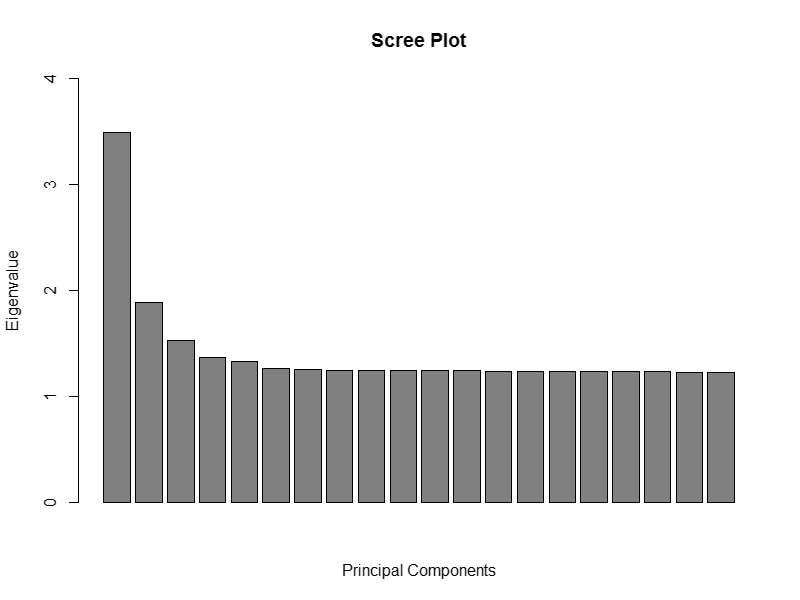

Supplement: Additional file 1: Table S1 — SNPs from top 1000 from our combined CAMH/IoP GWAS for BPAD, for which at least one other non-overlapping GWAS also shows association at same gene. Table S2: Top 68 SNPs (showing suggestive association to BD: p < 0.0001) in our combined (CAMH and IoP) GWAS. Table S3. Top 132 SNPs (showing suggestive association to BD in our CAMH family cohort: p < 0.0001). Table S4: Listing suggestive significant genic SNPs for combined Toronto and London GWAS for which there are 4 or more suggestive significant SNPs among the top 1000, and for which no other positive reports have been published to date Figure S1: Scree plot of principal components (PCs) of the genotypes in the case–control samples. [file 1471-2350-15-2-S1.docx]
